# Benchmarking 3D Structure-Based Molecule Generators

**DOI:** 10.1021/acs.jcim.5c01020

**Published:** 2025-07-25

**Authors:** Natasha Sanjrani, Damien E. Coupry, Peter Pogány, David S. Palmer, Stephen D. Pickett

**Affiliations:** † Department of Cheminformatics, Research Technologies, 1929 GSK, Gunnels Wood Road, Stevenage SG1 2NY, U.K.; ‡ Department of Pure and Applied Chemistry, 3527 University of Strathclyde, Thomas Graham Building, 295 Cathedral Street, Glasgow G1 1XL, U.K.

## Abstract

To understand the benefits and drawbacks of 3D combinatorial and deep learning generators, a novel benchmark was created focusing on the recreation of important protein–ligand interactions and 3D ligand conformations. Using the BindingMOAD data set with a hold-out blind set, the sequential graph neural network generators, Pocket2Mol and PocketFlow, diffusion models, DiffSBDD and MolSnapper, and combinatorial genetic algorithms, AutoGrow4 and LigBuilderV3, were evaluated. It was discovered that deep learning methods fail to generate structurally valid molecules and 3D conformations, whereas combinatorial methods are slow and generate molecules that are prone to failing 2D MOSES filters. The results from this evaluation guide us toward improving deep learning structure-based generators by placing higher importance on structural validity, 3D ligand conformations, and recreation of important known active site interactions. This benchmark should be used to understand the limitations of future combinatorial and deep learning generators. The package is freely available under an Apache 2.0 license at github.com/gskcheminformatics/SBDD-benchmarking.

## Graphical abstract

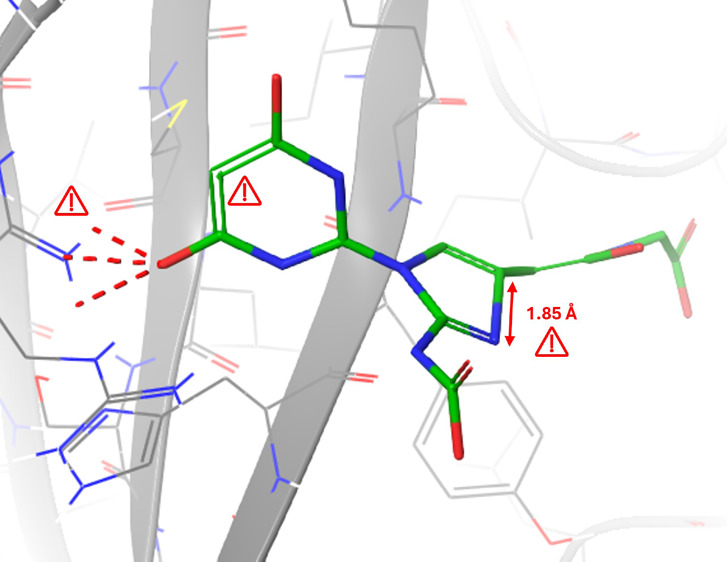

## Introduction

### The Role of Molecule Generators

The decline in the discovery of new drugs, as described by Eroom’s law, has seen the number of new drugs approved per billion US dollars spent on R&D halve every nine years since 1950, despite increased investment. A contributing factor is suggested to be the saturation of commercially crowded therapeutic areas^1^ coupled with the inadequate exploration of vast chemical space, which is estimated to contain approximately 10^60^ potential drug-like molecules.^2^ Given the impracticality of searching this entire space for viable drug candidates, researchers focus on smaller, targeted regions for exploration and testing. Due to the high cost and slow pace of experimental testing, computational methods, such as 3D molecule generators, offer a promising alternative for efficiently navigating and exploiting chemical subspaces with the highest potential for success.

Molecular generators are categorized into ligand-based drug design (LBDD) and structure-based drug design (SBDD) methods. LBDD approaches generate novel compounds by extrapolating from known binders considering either 2D or 3D ligand information, whereas SBDD relies on the protein of interest to investigate binding site interactions and therefore incorporates 3D information on the ligand and the protein.^3^ Traditional knowledge-based or sampling techniques and generative Artificial Intelligence (AI) can be used in both methodologies. In generating new compounds, both LBDD and SBDD can operate through one-shot generation, forming the entire structure at once, or iteratively, building atom-by-atom or fragment-by-fragment.^4^ SBDD can specifically use 3D protein structures, which may be derived experimentally, via homology models, or through computational folding.^5^

#### Combinatorial Methods

Early works focused on breadth-first or depth-first methods to generate molecules and navigate the combinatorial chemical search space and are analogous to circular and linear molecular fingerprints. These methods find global solutions by considering all possible combinations of atoms (nodes) and bonds (edges) to generate molecules. Due to extremely large chemical search spaces, these methods are often used in conjunction with constraints such as using small sets of fragments and linkers instead of atoms and bonds.^3^ A popular choice for navigating this search space are Evolutionary Algorithms, such as Genetic Algorithms (GAs), based on the theory of evolution through the mechanism of survival of the fittest. Reproduction, mutation, and recombination operators are used on a set of parent molecules to generate children. A fitness function is used to assess which child molecules survive and are promoted to parents for the next rounds of generation. This process is repeated until a termination step is reached.^6^

To incorporate protein structure into these methods, the protein binding site is commonly used to extract useful receptor shape and ligand–receptor interaction information. Rule-based approaches such as HSITE find hydrogen bond regions on a receptor defined by an ideal geometry derived from known crystal structures, with subsequent methods identifying other covalent and noncovalent interactions. Grid-based approaches such as GRID divide the receptor into a set of grid points and place different probes at each point for the computation of interaction energies that aid in understanding important ligand-protein interactions at each point.^3^ These interaction points are then used to sample atoms or fragments and evaluated within the active site. The qualities of generated compounds are assessed by approximating the binding free energy between a ligand and receptor explicitly through expensive MD calculation methods (such as FEP), empirical scoring functions consisting of learned weights for known terms such as hydrogen bonding, or knowledge-based scoring functions based on ligand-protein interactions extracted from crystal structure databases.^4^ AutoGrow4 ^7^ and LigBuilderV3 ^8^ are two open-source GAs that combine known drug-like fragments and assess the quality of generated compounds in the active site through docking and empirical scoring functions, respectively.

#### Artificial Intelligence

AI is the study of machines that mimic human cognition and can thereby simulate human intelligence. Discriminative models are those that use a learned distribution to make predictions on new data points whereas generative models, broadly a branch of Deep Learning (DL), use a learned distribution to sample new data points. The main distinction between combinatorial methods and DL generative models is that the former relies on defined rules and scoring functions for molecule generation. Instead, DL models use a data-driven approach to learn complex nonlinear relationships to infer rules. The majority of DL generative models explicitly model a continuous chemical space that can be sampled from, unlike combinatorial methods which use search methods to navigate combinatorial chemical space. DL generative models can be seen as more generalizable and avoid the bias of user-defined rules or scores, although direct comparisons of these methods have thus far shown inconclusive results.^9^

Recently, there has been a shift toward the field of Geometric Deep Learning which aims to rectify the Curse of Dimensionality (CoD) when faced with high dimensional data through the addition of inductive biases.^10^ The CoD refers to the need for exponentially more data as the data dimensions (features) increase since sufficient training points are needed with different combinations of features to enable model learning. The introduction of inductive biases is achieved through the consideration of the underlying geometric “domain” of the data, for example, molecules can be described in the graphical domain and images can be described in the grid domain. Symmetries of the underlying domain are embedded in the model to reduce the space of possible functions the model can learn, making higher dimensions more manageable.^11,12^ *Invariant* neural networks produce the same predictions if applied to a molecule or a molecule with a symmetry group operation applied to it. While *equivariant* neural networks produce a prediction change if applied to a molecule or a molecule with a symmetry group operation applied to it.^12^ For example, SE­(*n*)-equivariant networks would produce different predictions if the set of translations and rotations from the SE­(*n*) symmetry group were applied to the same inputs. This makes intuitive sense for ligands modeled in protein receptors as a molecule translated a few Angstroms away from the receptor would remove important contacts in the binding site.

A variety of different DL architectures have been explored for both 2D and 3D molecule generation. Variational AutoEncoders (VAEs) and Recurrent Neural Networks (RNNs) were first used for the de novo generation of SMILES strings. However, due to their complexity and nonuniqueness, there were issues with the generation of valid molecules.^13^ The easier grammars of SELFIES and the use of fragments showed the improved generation of valid molecules.^14–18^ Graph-based generation also showed improvements in validity, however, their sequential generation does not generalize well to larger molecules.^19–22^ Generative Adversarial Networks (GANs) have been explored for SMILES and graphs, however, are notoriously difficult to train and do not show improvements compared to existing VAE, RNN, and graph network methods.^23–31^

3D structure-based generation has more recently been explored to optimize 3D properties explicitly rather than implicitly through 1D and 2D methods. For example, Pocket2Mol^32^ uses a sequential atom and bond generation approach using GNNs taking the protein active site and ligand as input. Newer model architectures such as normalizing flows and diffusion models have also been explored.^32–45^ Chirality can also be embedded into the design of models, for example by using SO(3)-equivariant networks, rather than through postprocessing steps.^46,47^ For example, DiffSBDD uses a diffusion model created with SO(3)-equivariant networks to generate molecules in a one-shot manner, predicting bonds as a postprocessing step with OpenBabel.^46^

Current limitations of 3D de novo design include computational load, issues with generating synthesizable molecules, and lack of experimental validation of current methods.^48^ Recently, methods have been developed that have been shown to improve the chemical validity of generated compounds. For example, PocketFlow improves on sequential generation through its bond predictor with explicit knowledge guidance and resamples atoms and bonds if unreasonable bonds are predicted.^45^ MolDiff, although not a structure-based generator, is a diffusion model that uses different noising strategies for atoms and bonds and includes bond guidance into its atom position predictor, showing better validities and 3D conformations of generated compounds.^49^ MolDiff was used with a pharmacophore and protein masking strategy for structure-based generation, named MolSnapper, and was shown to perform similarly to DiffSBDD^46^ for interaction recreation, while generating an increased fraction of physically viable compounds.^50^ Interestingly, when comparing commonly reported benchmarking metrics reported across several key papers, it is not clear that any one model architecture outperforms the others (Figure SI 1). Instead, the metrics improve and plateau as the field develops and gains experience, indicating that commonly reported metrics are not sufficient for evaluating 2D or 3D molecule generators, and requires the development of more challenging benchmarks. Unfortunately, there is currently no unified or standard benchmark for 3D de novo generation methods although progress is currently being made in this area.^51–56^ For 3D generation, evaluations on conformations of molecules in terms of bond lengths, bond angles, and torsion angles in search of the bioactive conformation are important. RMSDs have been previously used, however, can be sensitive to the molecular weight and the size of molecules.^57^ Structure-based design methods require additional evaluations on active site interactions.

### Data Sets & Benchmarks

#### Data Sets and Biases

Common data sets used for training and benchmarking generative models can be divided into compound-only, ligand-protein, and combined data sets. Among the compound data sets, ChEMBL^58^ and ZINC^59^ are prevalent. ChEMBL provides information on binding constants and chemical properties, while ZINC offers synthesized or purchasable compounds frequently employed for docking-based virtual screening. On the other hand, the Directory of Useful Decoys, DUD, data sets (including DUD-E and DUDEZ) serve as combined data sets featuring 102 cocrystal structures containing active compounds and property-matched decoys sourced from ChEMBL.^60,61^ Although commonly used for virtual screening enrichment scoring, these property-matched decoys have been shown to contain unrealistic structures.^60^ Differences in molecular properties, such as hydrogen-bond donor counts, between actives and decoys make them easily distinguishable. To address these issues, the LIT-PCBA data set was developed, drawing from the PubChem BioAssay database to include known actives and inactives without generating artificial property-matched decoys.^62^

In terms of ligand-protein data sets, the Protein Data Bank (PDB) offers experimentally determined crystal structures of proteins and their complexes.^63^ The sc-PDB data set^64^ refines this by focusing on complexes with ligandable binding sites, though its limited sample size and ligand diversity can lead to model overfitting when used for training molecule generative models.^65^ For 3D structure-based generators, data sets like BindingMOAD^66^ (which is now no longer updated) and PDBBind^67^ are widely used, as they curate binding affinities from real cocrystal structures from the PDB database and apply specific filtering criteria. PLINDER^68^ defines pocket-based splits to reduce data leakage and BioLiP2 ^69^ curates the PDB by extracting biologically relevant protein–ligand interactions. PLINDER and BioLiP2 are larger than other ligand-protein data sets as they do not curate binding affinities. Nonetheless, the CrossDocked2020 data set is perhaps the most extensively used for training structure-based generators. It is an expansion of PDBBind by cross-docking virtual ligands from known and structurally similar cocrystal structures into other protein structures with comparable binding pockets, thereby increasing data set size and diversity.^70^ However, this approach presumes that a ligand’s binding affinity remains consistent across similar receptors, an assumption that could be overly simplistic. Biased docking scores were generated compared to the curated data in BindingMOAD, likely due to training on docked structures.^46^ A comparison of PDB entries across PDBBind, CrossDocked2020, and BindingMOAD (see Table 1) reveals that CrossDocked2020 shares the fewest PDBs with PDBBind, while BindingMOAD encompasses many PDBs present in the other data sets.

**Table 1:** Intersection of PDB IDs in the BindingMOAD, CrossDocked2020, and PDBBind v2016 Datasets, where the BindingMOAD Set Has Been Filtered to Remove Carbohydrate and Peptide Ligands

	BindingMOAD	CrossDocked2020	PDBBind
BindingMOAD	16,609		
CrossDocked2020	5014	20,903	
PDBBind	6905	1801	19,120

#### Benchmarks

In recent years, efforts to evaluate de novo generative models have been hampered by inconsistencies in evaluation metrics and data sets or subsets used. To address this challenge, several benchmarks have been developed to provide a more structured and standardized evaluation framework. Among these, MoleculeNet^71^ is notable for addressing quantum mechanics, physical chemistry, biophysics, and physiology data sets. The latter three are particularly significant for drug design, as they encompass both compound-only and protein–ligand data. Baseline conventional and graph-based models, such as logistic regression and graph convolutional networks (GCNs), were implemented using the DeepChem package with a variety of 2D featurization schemes. However, when applied to protein–ligand binding affinity prediction using PDBBind data,^67^ these models were susceptible to overfitting due to the limited data set size and the temporal split used for training, validation, and testing.^71^

GuacaMol and MOSES were introduced as 2D benchmarks focusing on the validity and novelty of compounds generated for ligand-based drug design (LBDD), offering standardized evaluation metrics and baselines.^72,73^ GuacaMol leverages a filtered ChEMBL24,^58^ whereas MOSES uses a filtered ZINC Clean Leads library.^59^ Note that MOSES is both the name of the benchmark and the filtered ZINC Clean Leads Library data set here. The GuacaMol authors demonstrated that state-of-the-art models could readily optimize properties such as log*P* and various distribution metrics like validity, novelty, and uniqueness (as we also observed in Figure SI 1).

In response to these challenges, more robust benchmarks have emerged. Therapeutics Data Commons (TDC) provides a platform for both LBDD and structure-based drug design (SBDD), employing data sets from ZINC, ChEMBL, and MOSES for LBDD, and data sets like PDBBind, DUD-E, scPDB, and CrossDocked2020 for SBDD. TDC defines metrics related to docking, synthesizability, and property performance, as well as receptor-specific predictions for GSK3β, DRD2, and JNK2.^74^ Furthermore, DOCKSTRING was designed with docking scores for 58 diverse targets, primarily selected from the DUD-E set. This benchmark evaluates docking score tasks, promiscuity of ligand binding to PPAR receptors, and selectivity in binding to JAK2 and LCK.^75^ Although this benchmark uses extensive kinase structure training data, making the task easier, the tasks reveal foundational insights into ligand selectivity and promiscuity. Tartarus builds on TDC data and uniquely includes compute-time evaluations for both training and inference.^76^ As described previously, the ChEMBL, DUD, PDBBind, and CrossDocked2020 data sets used in these benchmarks have been criticized for overfitting and bias.^46,60,71,72^ Historical analyses have shown that docking algorithms, while effective at reproducing ligand conformations, struggle with target-type dependencies and sometimes fail to accurately identify optimal binding poses.^77^ Nevertheless, docking scores have been proposed as the main baseline improvement for structure-based over ligand-based scoring functions, the latter of which are biased toward known drug-like fragments and properties.^78^ To critically assess models like ChemVAE, GrammarVAE, and REINVENT, a docking benchmark using known targets from ChEMBL (such as 5-HT1B, 5-HT2B, ACM2, and CYP2D6) was employed. However, it was found that ZINC compounds performed better in terms of docking scores.^79^ This highlights potential biases in generative methods that rely on docking scores and underscores the importance of additional criteria for evaluating SBDD models. In particular, metrics evaluating key interaction site reconstruction, RMSD to crystal poses, and binding affinities offer a more comprehensive assessment.

Specialized benchmarks like Atom3D^80^ and Durian^53^ have been developed to evaluate 3D inputs and generative models. Atom3D assesses models’ abilities to predict binding using 3D inputs derived from PDBBind while Durian evaluates property distributions, docking scores, and the overall quality of poses generated by models like LiGAN, Pocket2Mol, DiffSBDD, and others. Meanwhile, PoseBusters^51^ and PoseCheck^52^ provide insights into the chemical validity and interaction fidelity of generated 3D poses, emphasizing physiological relevance and energetics. Additionally, GenBench3D introduced a new 3D validity metric, focused on ligand conformation quality based on the Cambridge Structural Database (CSD).^54^ Finally, POKMOL-3D combined 2D and 3D benchmarks to assess various 3D structure-based generators, comparing them to the conventional method REINVENT.^56^ Building on previous findings,^54,55^ POKMOL-3D further demonstrated the suboptimal quality of many generated 3D conformations. These benchmarks emphasize the importance of additionally evaluating generative models within the context of traditional combinatorial methods.

Building on these benchmarks, this study introduces tests that are important for evaluating common tasks performed during the drug discovery process. Namely, the ability of molecule generators to recreate protein–ligand interactions known to produce downstream efficacy, the similarity of generated compounds to high-throughput screening hits, and the overall ability of generators to perform on unseen targets with good 3D conformations. Through this study and others like it, the goal is to understand the advantages and limitations of both deep learning and traditional methods in advancing molecular generation benchmarks.

## Methodology

A benchmark was developed and evaluated on four deep learning (Pocket2Mol,^32^ PocketFlow,^45^ DiffSBDD,^46^ and MolSnapper^50^) and two combinatorial generators (AutoGrow4 ^7^ and LigBuilderV3 ^8^) to assess their 3D *de novo* design capabilities across a range of selected tasks and targets from the BindingMOAD data set and two other virtual screening data sets.

### Data Set

The deep learning (DL) models Pocket2Mol, PocketFlow, and DiffSBDD were retrained on the BindingMOAD data set, excluding test proteins. Although CrossDocked2020 was shown to yield higher docking scores than BindingMOAD, it was excluded from training due to producing unrealistic protein–ligand interactions. The BindingMOAD data set was chosen for its high-quality, curated experimental structures and overlap with other data sets (Table 1). To filter structures from BindingMOAD, the scheme from DiffSBDD was adopted where all ligands with a QED (quantitative estimation of drug-likeness score) above 0.3 were kept. Additionally, lists of monosaccharides provided by the PDB and amino acids (standard and modified) were used to remove any structures containing peptides or oligosaccharides as their only ligands.

BindingMOAD labels its structures according to Enzyme Commission Numbers (ECNs) that classifies proteins into reactions they catalyze.^66^ Prior to splitting the data into train and test sets, 20 protein targets were selected which were not part of any other ECNs (Table SI 1). Each protein was selected to test whether, for each case, factors important for binding and potency such as interactions with important cofactors, impact of protein conformation, and binding site detection were considered by molecule generators.

The remaining BindingMOAD protein–ligand structures were then split on ligand chemotype (to ensure low bias in ligand structures for training and test) in an 80:20 train to test ratio resulting in 41,703 training complexes and 10,272 test complexes. Morgan fingerprints with a radius of 2 and RDKit’s MinMaxPicker algorithm were used to select complexes for each set based on compound similarity. To further characterize the train/test splits, Figure SI 2 shows how similar the distributions of some RDKit-calculated^81^ chemical properties (molecular weight, synthetic accessibility score, logP, and tPSA) of the training and test sets are, suggesting a reasonable ligand split, and the distributions of BindingMOAD ECNs and mmseqs2 clusters (obtained from the PDB at 90% sequence identity and mapped to BindingMOAD PDB IDs) in Figure SI 3 show reasonable sampling per cluster for the splits.

### Benchmark

This benchmark is split into 3 main tasks which are described below where the key evaluation metrics rely on the percentage of important active site interactions recreated (Hydrogen bond, hydrophobic, and salt bridge interactions were calculated with the Protein–Ligand Interaction Profiler^82^). It should be noted that this benchmark is not exhaustive and readers are recommended to manually check any compounds generated using molecule generators as part of their own evaluations and use the additional analysis that was performed in this study as a guide toward understanding model biases.

#### Filtering Schemes

The PoseBusters test suite is initially used to assess clashes between the input protein and generated ligands.^51^ Additional checks on the quality^83^ of generated compounds are included through filter checks from MOSES based on pan-assay interference compound (PAINS)^84^ and defined custom medicinal chemistry filters (MCFs).^73^ An allene SMARTS filter was created and fused ring filters from Walters and Murcko^85^ were used for additional checks (Table SI 4) as some allenes were observed for DL molecule generators. It should be noted that these filters were applied before task evaluations.

#### Task 1: Blind Set

As described previously, 20 targets were selected, one per singular ECN, to create the blind set to check for interactions important for potency. Pass/fail metrics based on similarity to known inhibitors, recreation of interactions for at least 50% generated compounds, and whether inclusion of important metals and cofactors change the distance of said compounds to these groups. Detailed pass/fail metrics are provided in Supplementary Table SI 1. Tables SI 5, 8, 11, 14, 17, 20 list the percentage of compounds passing each subtask for different methods, while average pass rates are summarized in Table SI 23.

#### Task 2: Target-Specific Checks

Bespoke pass/fail tests have been written to assess the ability to recreate important intermolecular interactions in at least 50% of generated compounds for known selective (ITK, LCK, AurB) and pan-active (JAK1, JAK2, JAK3, TYK2, BRD2, BRD3, BRD4, BRDT) targets (Table SI 2). Tables SI 6, 9, 12, 15, 18, 21 list the percentage of compounds passing each subtask for different methods, while average pass rates are summarized in Table SI 23.

ITK (interleukin-2-inducible T-cell kinase) and AurB (aurora kinase B) proteins were chosen to investigate the generation of molecules for the generic kinase domain, whereas LCK (lymphocyte-specific protein tyrosine kinase) was used to investigate the less frequently studied SH2 domain. The main protein residues important for interactions in ITK and AurB are Met438 and Ala157, whereas the main difference causing selectivity is an important mutation from Val419 in ITK to Leu138 in AurB. This is due to the space inside the binding pocket of the key hinge region changing, as seen in Figure 1.^86^

**Figure 1:**
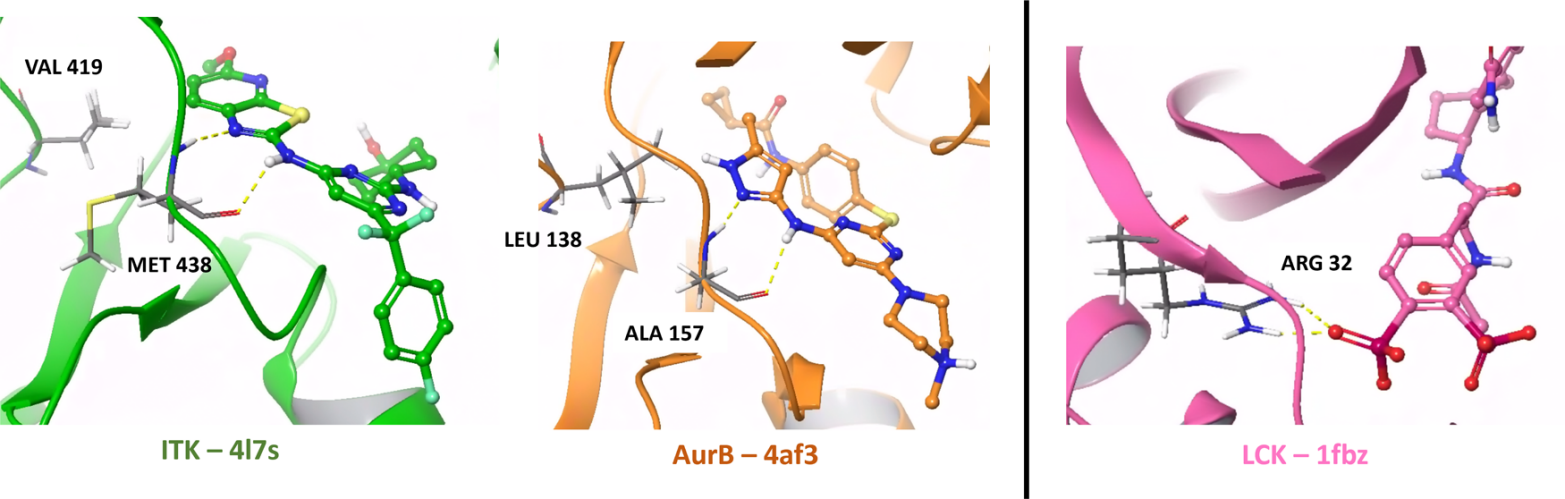
Selected PDBs of the proteins ITK, AurB, and LCK with key hinge interactions shown for ITK and AurB and an interaction in the SH2 domain shown for LCK.

Pan-activity was assessed for the JAK (janus kinase) and BET (bromodomain and extra-terminal domain) proteins. Hinge hydrogen-bond interactions are necessary for JAK activity as seen in Figure 2.^87^ As seen in Figure 3, BET proteins require interactions with a conserved Asn156 residue to elicit activity and for further potency, interactions with a hydrophobic shelf (the “WPF” motif), and bulk toward narrow “ZA” channel are required.^88^

**Figure 2:**
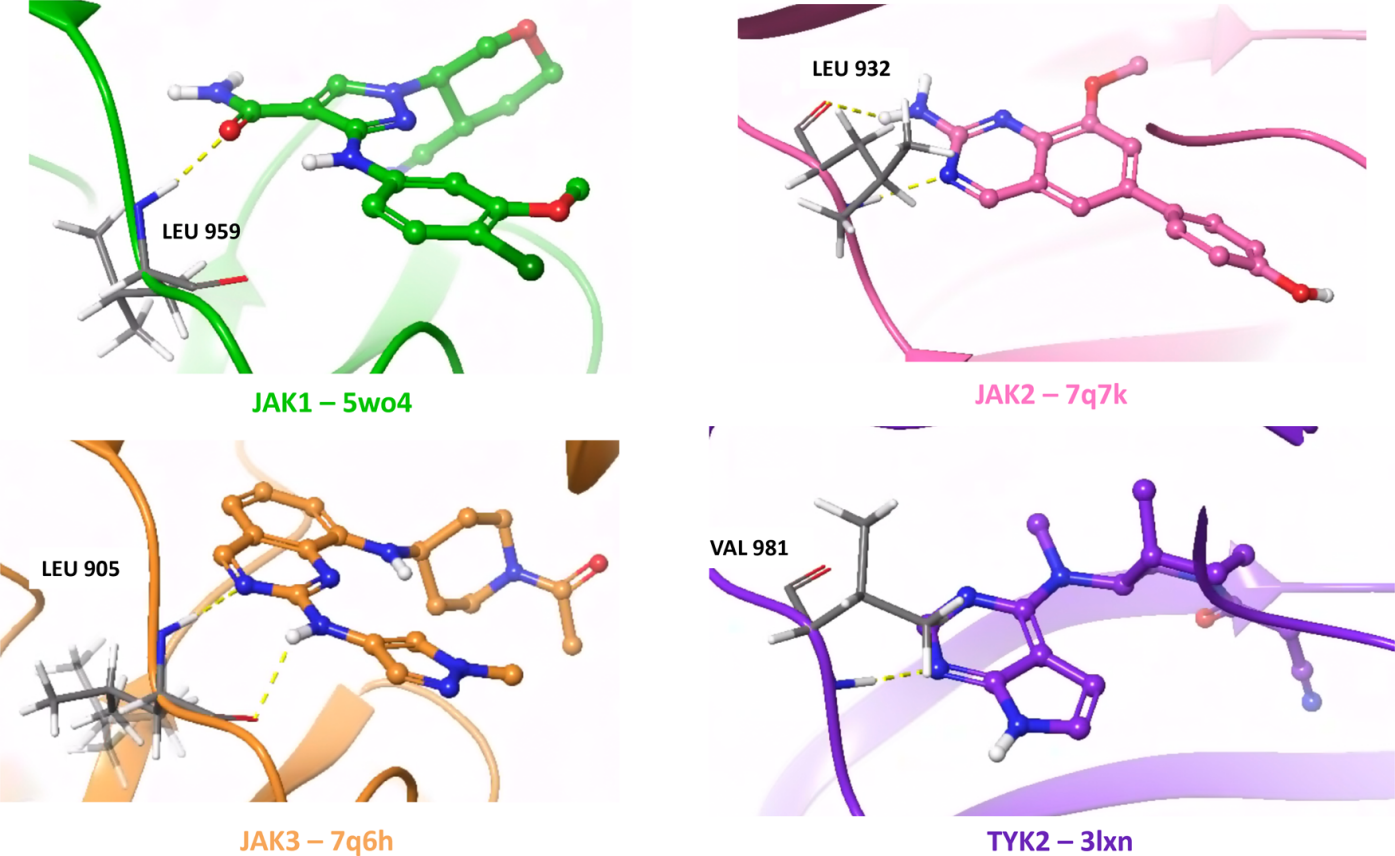
Selected PDBs of the proteins JAK1, JAK2, JAK3, and TYK2 with key hinge interactions shown.

**Figure 3:**
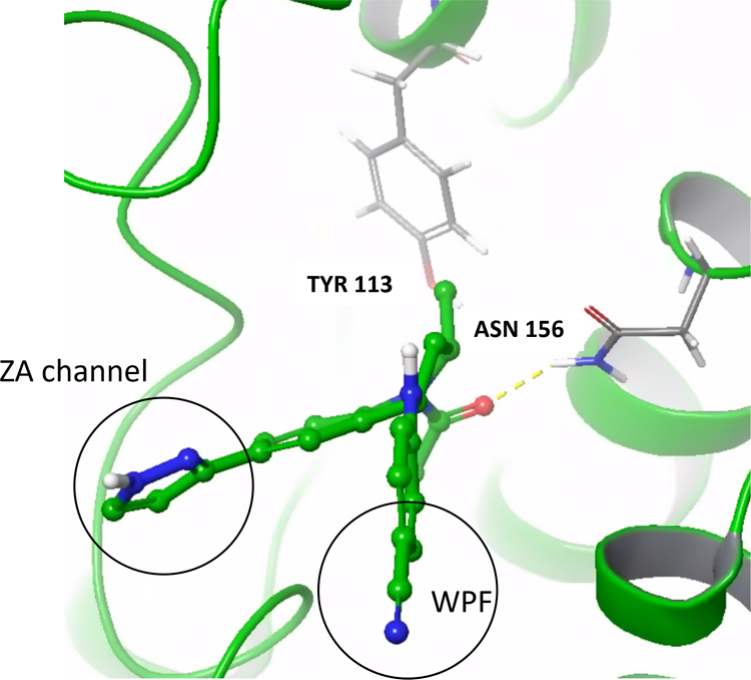
BRD2 PDB 6ddi shown with its crystallized ligand to illustrate the key interactions required for activity and potency.

An additional shikimate kinase protein was checked for interaction recreations for residues Asp34 and Arg58 as seen in the liGAN evaluation.^36^

#### Task 3: Virtual Screening

The COVID-19 Moonshot^89^ and CSAR 2014 ^90^ data sets were used to assess whether generated compounds were similar to hits identified in these studies, akin to a virtual screening workflow (Table SI 3). Pass/fail metrics were defined by recreation of interactions for at least 10% of generated compounds and an average Tanimoto similarity higher than 0.5 with hit compounds. The lower 10% cutoff was chosen to balance the trade-off between interaction recreation (exploitation) and searching novel chemical space (exploration) where more focus is usually placed on exploration for virtual screening. The 0.5 Tanimoto cutoff was chosen to distinguish between generated molecules and known synthesized compounds (Figure SI 5). These cut-offs are configurable in the code made available in the GitHub repository. It should be noted this analysis is limited as there were no negative examples for comparisons of dissimilarity. Tables SI 7, 10, 13, 16, 19, 22 list the percentage of compounds passing each subtask for different methods, while average pass rates are summarized in Table SI 23.

The COVID-19 Moonshot project was developed to enable research institutions and industrial collaborators to work toward finding a cure for the disease. The COVID-19 main protease, *M*_pro_, was used from the Moonshot set to assess generators. The CSAR 2014 data set contains published GSK in-house crystal structures and binding affinity data for the last Community Structure–Activity Resource (CSAR) exercise used to evaluate docking methods. It consists of the proteins FXa (coagulation factor Xa), SYK (spleen tyrosine kinase), and TrMD (S-adenosyl methionine-dependent methyl transferase).

#### Additional Analysis

To assess the biases of each model, visual assessments of ligand/pocket interactions and volume were conducted on samples using Schrodinger’s Maestro suite.^91^ The synthesizability of compounds is assessed using the normalized spacial score (nSPS)^92^ and similarity to a database of available synthesized compounds.

The feasibility of conformations of compounds was checked through the Mogul suite provided by CCDC^93,94^ and molecular relaxation using Embrace minimization in Maestro. Default minimization settings were used in Embrace: OPLS4 force field, water as the solvent, the PRCG gradient-based optimization method was used for a maximum of 2500 iterations with a convergence threshold of 0.05. Additional property metrics for the generation of drug-like compound distributions were calculated using MOSES^73^ and RDKit^81^ (synthetic accessibility score, molecular weight, drug-likeness QED, and log*P*) and analyzed.

### Generators Evaluated

Pocket2Mol,^32^ PocketFlow,^45^ and DiffSBDD^46^ were retrained on the BindingMOAD data set training and test splits outlined above. The same hyperparameters from the DiffSBDD all-atom BindingMOAD model were used whereas Pocket2Mol’s and PocketFlow’s CrossDocked2020-trained model hyperparameters were used for their retraining. Pocket2Mol, PocketFlow, and DiffSBDD loss and reconstruction errors curves, respectively, can be seen in Figure 4 where losses for Pocket2Mol have been smoothed by plotting the mean loss every 5000 iterations.

**Figure 4:**
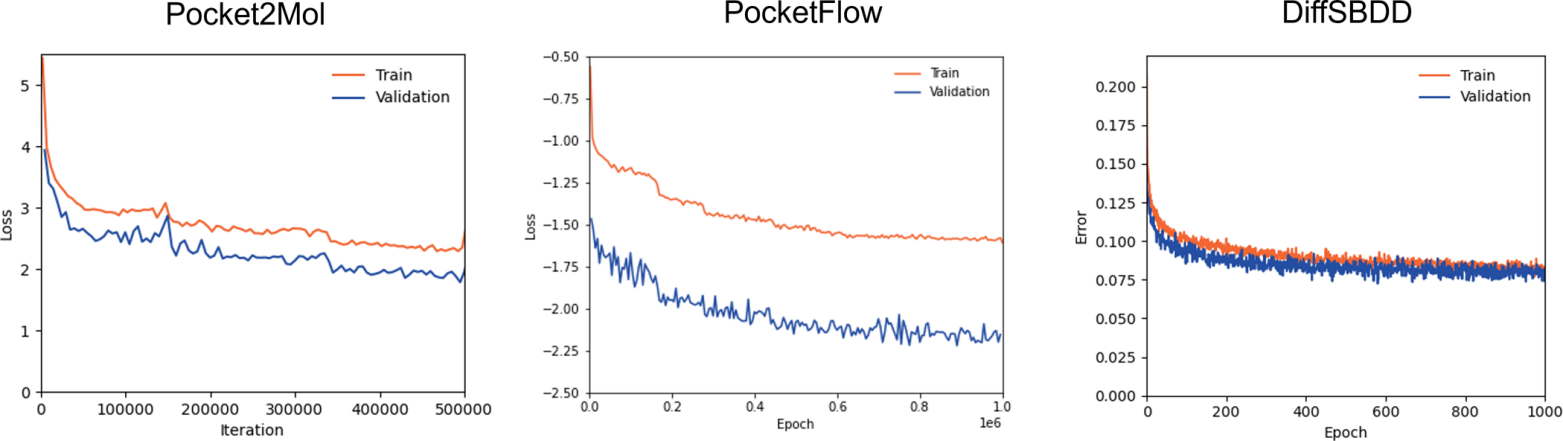
(Left) Pocket2Mol train and validation loss curves per iteration of sequential molecule generation. (Middle) PocketFlow train and validation loss curves per epoch. (Right) DiffSBDD train and validation reconstruction error curves per epoch.

During inference, each generator was queried to produce 10,000 molecules per PDB. Default parameters were used for both AutoGrow4 and LigBuilderV3 where only one run, rather than multiple short runs, were used to generate molecules per PDB. Summarized parameters can be found in the descriptions below and further details in the Supporting Information.

#### Pocket2Mol

Pocket2Mol uses an autoregressive technique to sample atoms and bonds sequentially from an *E*(3)-equivariant neural network until a full molecule has been created.^32^ Pocket2Mol was retrained using the BindingMOAD data set with the split outlined previously. The same network architecture was used and same hyperparameters as in the original paper, namely, a batch size of 8, an initial learning rate of 2 × 10^–4^, a learning rate decay of 0.6 when the validation loss does not increase for 8 iterations, for 500,000 total training iterations.

#### PocketFlow

PocketFlow uses a similar sequential generation process to Pocket2Mol, but with an SO(3)-equivariant Geometric Double Bottleneck Perceptron where the atom and bond prediction network are defined by normalizing flows and the bond predictor is guided using chemical knowledge to resample atoms and bonds if predicted bonds are not reasonable.^45^ PocketFlow was fine-tuned for 1,000,000 iterations with the BindingMOAD data set split used in this benchmark with the same ZINC pretrained network from the original study. The same hyperparameters were used as in the original paper: an initial learning rate of 2 × 10^–4^, a decay rate of 0.6, patience of 10, and a minimum learning rate of 1 × 10^–5^.

#### DiffSBDD

DiffSBDD uses an SO(3)-equivariant conditional diffusion model that can sample atom positions and types but bonds are inferred using OpenBabel. The method also has an “inpainting” ability to learn a joint distribution of protein and ligand atoms for lead optimization, however, this is not explored further in this evaluation.^46^ Similar to Pocket2Mol, DiffSBDD was retrained on the BindingMOAD data set with the split defined in this study. The same hyperparameters as in the original training were used from the all-atom BindingMOAD-trained network, namely, a learning rate of 5 × 10^–4^, a weight decay of 1 × 10^–12^, 500 diffusion steps, ligand-pocket edges defined by anything closer than 7 Å and pocket edges defined by anything closer than 4 Å, trained for 1000 epochs.

#### MolSnapper

MolSnapper,^50^ an E(3)-equivariant neural network, is based on the MolDiff^49^ diffusion model that was originally trained on the GEOM-Drug 3D ligand-only data set. This method required no retraining as pocket conditioning is performed during inference. The ground-truth crystal ligands were used to extract pharmacophores for input into the model.

#### AutoGrow4

AutoGrow4 uses a genetic algorithm (GA) and a docking-based fitness function to generate compounds using fragments. The program can be used for both de novo and lead optimization tasks, however, the latter is not considered in this evaluation.^7^ For sampling, a default box size of 23 × 23 × 23 Å^3^ was defined, centered on the crystal ligand’s center of geometry using the centering parameters, to allow a better comparison with Pocket2Mol’s default box size. Ten mutation and crossover operations performed for 10 generations and 5000 mutation and crossover operations performed for the last generation to sample 10,000 compounds per input protein. In certain cases this last generation did not finish running, therefore compounds were sampled from previous generations to be evaluated. Input fragments were taken from the default filtered ZINC library containing fragments of molecular weights between 100 and 150 Da and the Vina docking protocol defined in AutoGrow4 was used. It should be noted that the authors recommend using a larger number of independent runs with fewer generations as this is less likely to generate compounds that try to maximize the fitness function, however, this was not evaluated in this study to preserve a better comparison with LigBuilderV3.

#### LigBuilderV3

LigBuilderV3 is a GA, similar to AutoGrow4, that is composed of a Cavity step and a Build step.^8^ The Cavity step was used in the default detection mode around the input ligand, including metal atoms and water molecules. The Build step was defined for a single target with default molecular property ranges and no specific intermolecular interactions defined. The default of 9 stages were run to generate 10,000 compounds per input protein. As described later, LigBuilderV3 generated a large number of compounds per PDB, which had to be randomly subsampled for evaluation due to memory constraints.

## Results

The key issues with the compared generators are covered in depth in this section. More information on passes and fails for each method can be found in the Tables SI 5–SI 23. Overall, as seen in Table 2, when evaluating the generators on the proposed benchmark, every generator fails most of the tests. Out of 20 possible Blind Set proteins (Task 1 tests), Pocket2Mol, DiffSBDD, MolSnapper, and AutoGrow4 pass 3 while PocketFlow passes 1 and the traditional combinatorial method LigBuilderV3 passes none. Out of the 4 possible Target-Specific Checks (Task 2 tests), PocketFlow, DiffSBDD, and MolSnapper passed 1 and Pocket2Mol passed 2 with the combinatorial methods passing none. Most methods pass the Virtual Screening Check (Task 3), as compounds need to have an average Tanimoto similarity of more than 0.5 with hit compounds and only 10% of the time interactions need to be recreated. The fingerprint used for similarity calculations is the substructure RDKit RDKFingerprint with a default size of 2048 bits. It might be unreasonable to expect such a high similarity to hits, particularly if chemical space is to be explored as unique and novel compounds will be dissimilar to known hits. A shape and color 3D similarity score, SC_RDKit_,^50^ was also tested with a 0.5 cutoff. This cutoff was chosen as MolSnapper was shown to have a Top-1 average of 0.721 in comparison to 0.417 from MolDiff.^50^ We therefore believe, for this virtual screening task, either similarity metric can be used as none of the methods showed additional passes as the main failure points were due to not enough compounds being generated or interactions recreated per protein.

**Table 2:** Fraction of Passed Tests per Task for Each Evaluated Method after Compound Filtering through MOSES, PoseBusters and Additional Allene and Fused Ring Checks^a^

	task 1	task 2	task 3
Pocket2Mol	3/20 (1069)	2/4 (857)	2/2 (8282)
PocketFlow	1/20 (32,500)	1/4 (37,500)	0/2 (240,000)
DiffSBDD	3/20 (21,261)	1/4 (32,306)	2/2 (215,106)
MolSnapper	3/20 (12,541)	1/4 (13,719)	2/2 (96,655)
AutoGrow4	3/20 (813)	0/4 (192)	1/2 (719)
LigBuilderV3	0/20 (5425)	0/4 (650,990)	0/2 (3,832,963)

a The required percentage of recreated interactions in 50% for tasks 1 and 2 and 10% for task 3. The total number of compounds generated post-filtering for each task are shown in brackets.

For the majority of Pocket2Mol, PocketFlow, DiffSBDD, MolSnapper, and AutoGrow4 failures, the reasons were the inability of the same crystal intermolecular interactions to be recreated for generated compounds more than 50% of the time whereas LigBuilderV3 failed to generate a sufficient number of compounds per PDB for analysis or failed to generate compounds at all during inference. The highest average interaction recreation percentage was observed for DiffSBDD, followed by Pocket2Mol and MolSnapper which had a similar performance (see Table SI 23).

### Chemical Validity

Pocket2Mol passed MOSES checks, on average, for 81% of generated compounds (Table 3). However, upon visual inspection, allene bonds were shown to be generated in a large number of cases. Therefore, an additional filter was created to remove allene bonds and certain fused rings defined by Murcko and Walters.^85^ This was shown to pass for the Blind Set test (Task 1), on average, only 44% of the time. The same conclusions were drawn for the Target-Specific and Virtual Screening tests (tasks 2 and 3), with the majority of issues arising from the chemical structures generated by the algorithm with allenes and fused rings. It was thought that this could be a result of under-training (Figure 4), therefore, Pocket2Mol was trained for 500,000 further iterations (Figure SI 8) and evaluated using the allene and fused ring checks. There was a 16% increase in the number of compounds passing these filters upon further training (Figure SI 9), however, the model trained on the CrossDocked data set still showed superior performance regarding this metric suggesting the data set size has an effect on performance. Unfortunately, although the chemical validity of generated compounds improved on further training, the same trend was not observed for the number of recreated interactions which decreased for some proteins (Figures SI 10–SI 12). This could be due to the generation of better structures and better 3D conformations, resulting in the filtering of strained compounds that generate active site interactions. However, it could also suggest overfitting on certain domains, such as the kinase domain, as shown in Figure 8. Overall, this suggests there is a balance between the model exploring 3D active site interactions and chemically valid structures.

**Table 3:** Percentage of Compounds, on a Random Sample of 1000, That Pass MOSES, PoseBusters and Additional Allene and Fused Ring Filters

	MOSES	allene & fused ring	PoseBusters	passed all checks
Pocket2Mol	81.2	43.5	48.2	40.4
PocketFlow	72.1	99.5	12.6	10.9
DiffSBDD	53.4	95.6	54.8	33.3
MolSnapper	49.0	83.7	24.7	15.1
AutoGrow4	35.6	99.9	92.7	32.5
LigBuilderV3	47.2	100.0	84.2	41.0

PocketFlow introduced knowledge guidance to avoid the generation of unreasonable bonds through explicit filters and resampling strategies.^45^ For example, Pocket2Mol showed a large number of allene bonds being generated, that were filtered in PocketFlow’s knowledge guidance framework by asking the generators to resample atoms and bonds. Unfortunately, as seen in Table 3, although most compounds passed MOSES and allene/fused ring checks, the majority of compounds failed on the internal energy and distance to protein PoseBusters checks, suggesting clashes in the active site. It was also shown that the compounds generated by PocketFlow were much smaller than those from other methods (Figure SI 6).

Approximately half of the compounds generated by DiffSBDD failed MOSES filters. Upon further inspection, this is due to the MOSES custom medicinal chemistry filters (MCFs), in particular, the 3-membered ring filters. This could be due to undertraining, although it was also shown by the authors of Pocket2Mol, that methods inferring bonds through OpenBabel generated a large number, around 30%, of 3-membered rings.^32^ For both Pocket2Mol and DiffSBDD, generating correct bonds is difficult. However, upon further assessment to understand whether these differences are due to the DL models or the bond prediction algorithms, the crystal structure ligands were taken from BindingMOAD, their bonds removed, and positions and atom types provided to OpenBabel. OpenBabel did not generate any 3-membered rings for crystal ligands if they were not already present in the original crystal structure. This shows that atom positions and types generated by DiffSBDD are likely too close, which could be due to bond information not being provided to the initial ligand embedding or due to the noising process of diffusion models changing atomic positions and therefore bond predictions. A bond prediction algorithm based on distances and angles taken from the analysis of the CSD, similar to that developed for PDB bond inference,^95^ will be important in alleviating these issues, whether considering sequential or one-shot generation of bonds.

MolSnapper uses the MolDiff algorithm, which was developed to avoid atom-bond inconsistencies in generated molecules, as atoms generated too close to one another lead to unreasonable bond predictions, as seen for 3-membered rings with DiffSBDD.^49^ Using the MolDiff algorithm within MolSnapper did not show better performance with regard to the MOSES filters where MolSnapper, similar to DiffSBDD, still generated a large number of 3-membered rings and charged compounds, suggesting the noising strategy in diffusion models affect bond prediction due to noisy atom position predictions.

Overall, all the deep learning methods showed lower pass rates on PoseBusters metrics (seen in Table 3), mainly due to steric clashes between generated compounds and the protein active site.

For AutoGrow4, less than half of the generated compounds passed MOSES metrics, failing on MCFs, in particular the oxirane SMARTS, and a defined charge filter which penalizes charged compounds. AutoGrow4 performs better on the PoseBusters metrics, suggesting better conformations generated due to the conformer generator and docking protocol used in the GAs fitness function.

LigBuilderV3 had difficulty generating compounds with many targets failing due to time limits or the Cavity step being unable to locate a sensible pocket for generation. However, when the Cavity step passed and time limit was not reached, the algorithm generated the most number of compounds per PDB compared to other methods. Again, almost half of the generated compounds fail on the MOSES filters with a lower pass rate than AutoGrow4 on PoseBusters metrics. The difficulty for combinatorial methods to pass the MOSES filters could indicate that these checks are too stringent for traditional generators and may require some adjustments, particularly to the penalization of charged atoms.

The feasibility of molecular conformers was assessed with the Mogul tool from the small molecule crystallographic database, CCDC.^93,94^ Mogul can search databases of known intramolecular geometries when compared to input molecules. Version 2024 was used on 50 random samples of compounds per PDB from each generative method, a plot of labeled “unusual” torsion angles, bond angles, and bond lengths can be seen in Figure 5 below, where “unusual” refers to values outside the predefined Mogul range. Although these were not flagged by PoseBusters, all the deep generative models show a higher number of unusual geometries when compared to the combinatorial methods. The combinatorial methods are based on fragments of known chemical structures and use rule-based methods to add bonds between fragments whose conformations are checked or docking is used, meaning geometries are more sensible.

**Figure 5:**
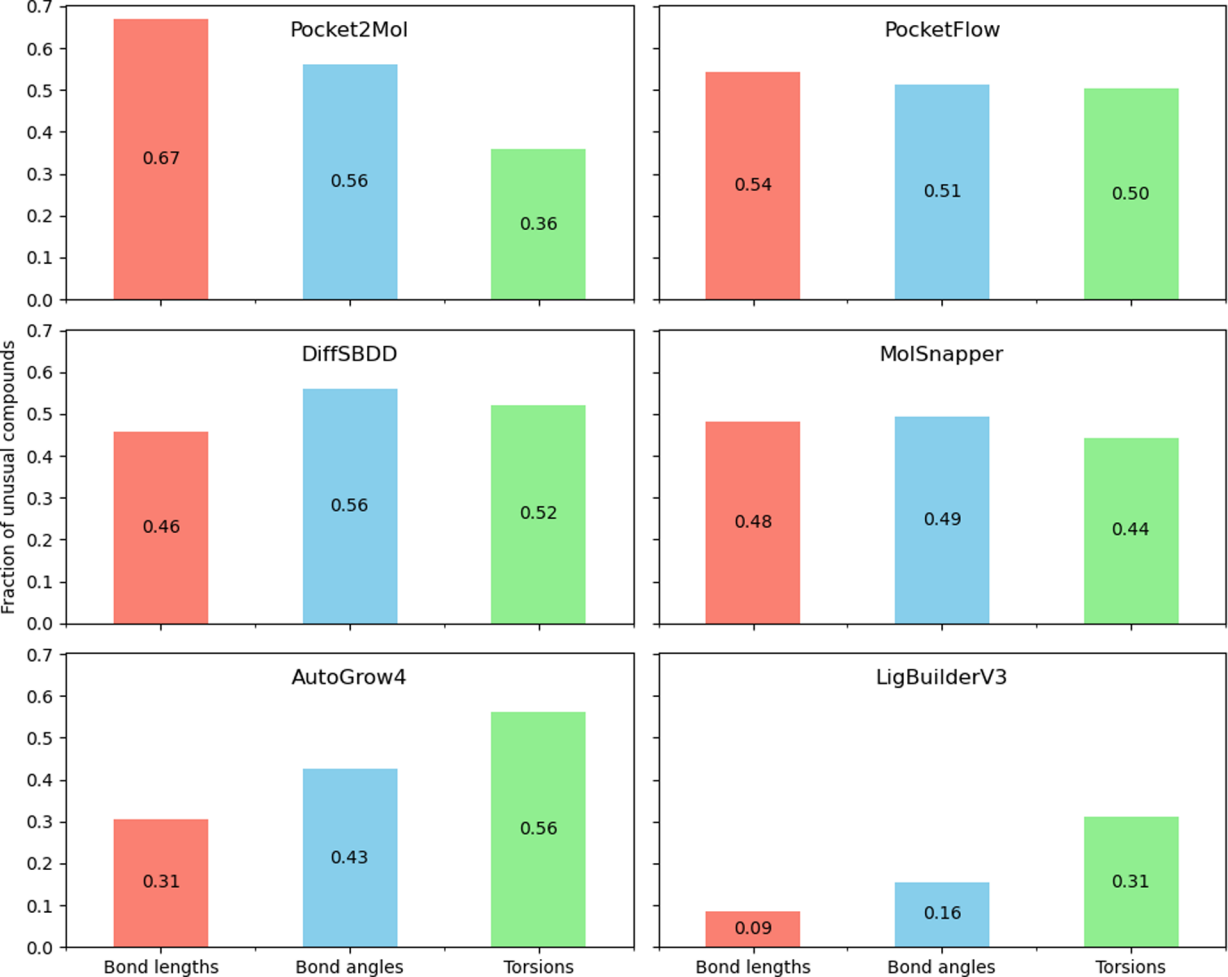
Mogul-defined unusual torsions, bond angles, and bond lengths of generated molecules compared to known CCDC and GSK data assessed with Mogul version 2024. The labels in each bar chart correspond to fractions of unusual geometries. Only annotations with the default 15 “‘enough hits”’ from Mogul were kept.

Additional molecular relaxation was performed using Schrodinger’s 2024.02 Embrace minimization method on a random sample of 150 compounds from the Virtual Screening task protein 4Q6R for each generative method where ligand atom positions are minimized and the protein is fixed.^91^ After minimization, the percentage of recreated interactions does not improve, suggesting the unrealistic conformations somewhat contributed to recreated interactions, in particular for MolSnapper where all recreations are lost postminimization (Table 4). The average RMSD change pre- and post-minimizations in Figure 6 show lower RMSDs for DiffSBDD and AutoGrow4 with slightly larger deviations seen for Pocket2Mol, LigBuilderV3, and PocketFlow. MolSnapper shows the largest changes to conformations post-minimization, further suggesting unrealistic conformations generated for this method despite the additional bond guidance during training. It should be noted that the RMSD of the original crystal ligand compared to the Embrace minimized conformer was 2.37 Å and is used to scale the axis here, the full distributions can be seen in Figure SI 13. Pocket2Mol RMSDs could be lower than expected because allene structures were corrected within Maestro to aromatic rings before Embrace minimization was performed.

**Table 4:** Average Percentage of Re-Created Interactions, Calculated Using PLIP,^82^ from Embrace Pre- and Post-Minimization, where the Percentage in Brackets Refers to Post-Minimization

	percentage hydrophobic recreated	percentage H-bond recreated
Pocket2Mol	28.0 (19.3)	12.0 (11.3)
PocketFlow	0.0 (0.0)	0.0 (0.0)
DiffSBDD	18.5 (13.9)	72.2 (51.4)
MolSnapper	1.8 (0.0)	89.3 (0.0)
AutoGrow4	37.7 (31.9)	51.4 (34.0)
LigBuilderV3	3.9 (6.5)	4.2 (3.5)

**Figure 6:**
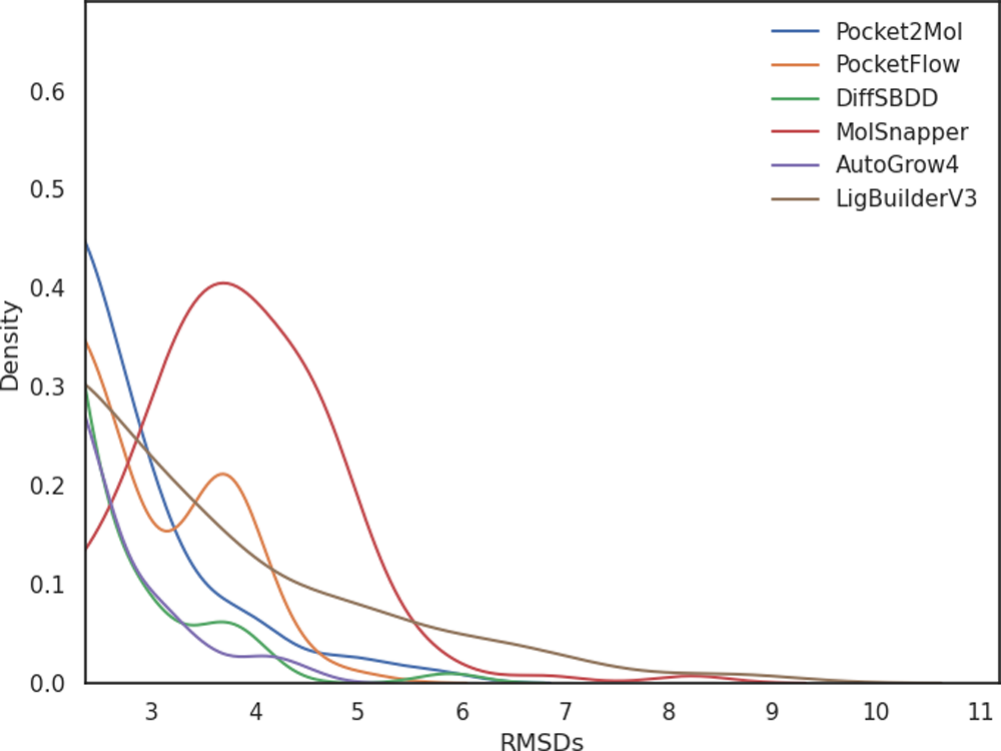
RMSDs, in Angstroms, pre- and post- Embrace minimization for each generative model. The crystal ligand RMSD of 2.37 Å has been used as a cutoff for the *x*-axis (the full distributions can be seen in Figure SI 13).

This geometry and intramolecular energy analysis further show that the deep generative models produce worse 3D conformations than the combinatorial methods.

In addition to conformations of combinatorial methods showing better alignment than DL methods to the CSD, further analysis of synthesizability (Figure SI 5) showed MolSnapper and LigBuilderV3 had greater similarities to known synthesized compounds whereas PocketFlow had low similarities. The property metrics show reasonable divergence for combinatorial methods in Figure SI 6, however, PocketFlow generated much smaller compounds (low molecular weight) compared to other methods and therefore had lower similarities to known synthesized compounds. Additionally, when assessing the molecular complexity score nSPS, it is possible that LigBuilderV3 might generate compounds that are too simplistic and does not explore chemical space as widely as other methods whereas AutoGrow4 generates more complex structures (Figure SI 7).

### Active Site Interactions

To assess active site interactions, proteins from the Target-Specific Checks (Task 2) were evaluated. Although LigBuilderV3 generates a large number of compounds when run (Table 2), each PDB required for the test did not have compounds generated for it. This is also indicated in Supplementary Table SI 23, where the standard deviation of the recreated fraction is greater or equal to the average. The tests require at least 100 compounds per PDB, after the filtering step, to ensure sufficient data is sampled for analysis. For example, in Task 2, LigBuilderV3 failed to generate any compounds for PDB 1FBZ (protein LCK for the ITK selectivity analysis) but generated sufficient compounds for the other two PDBs 4AF3 (AurB) and 4L7S (ITK).

An interesting observation was noted from the Task 2 evaluation for Pocket2Mol, where hydrogen bond interactions were recreated in the generic kinase domain for ITK and AurB (both >95%), but failed to recreate for the SH2 domain LCK (<30%, see Figure 7 left). Upon further inspection of domains it was found that the well-studied kinase domain was over-represented compared with the SH2 domain in both the train and test sets, appearing more than five times (Figure 8). This suggests further considerations for training splits, such as by pocket or protein domain, are required for Pocket2Mol and DiffSBDD particularly as a drop in performance was seen upon longer training (Figures SI 10–SI 12). DiffSBDD is also shown to have recreated a higher percentage of interactions in comparison to other methods for the BET task with MolSnapper performing worst across proteins in this task (Figure 7 center). Both of the diffusion models, DiffSBDD and MolSnapper, showed similar fractions of interactions recreated for the JAK task when compared to Pocket2Mol (Figure 7 right). LigBuilderV3 failed to generate compounds for LCK, BRD3, and BRD4, however, recreated some interactions with the other proteins showing a similar performance to Pocket2Mol for BRD2 (Figure 7 center). The lower fractions of interactions can be attributed to its wider exploration of the pocket (Figure 10). Finally, AutoGrow4 performs reasonably well and shows more consistent performance compared to LigBuilderV3 due to its lower failure rate during molecule generation.

**Figure 7:**
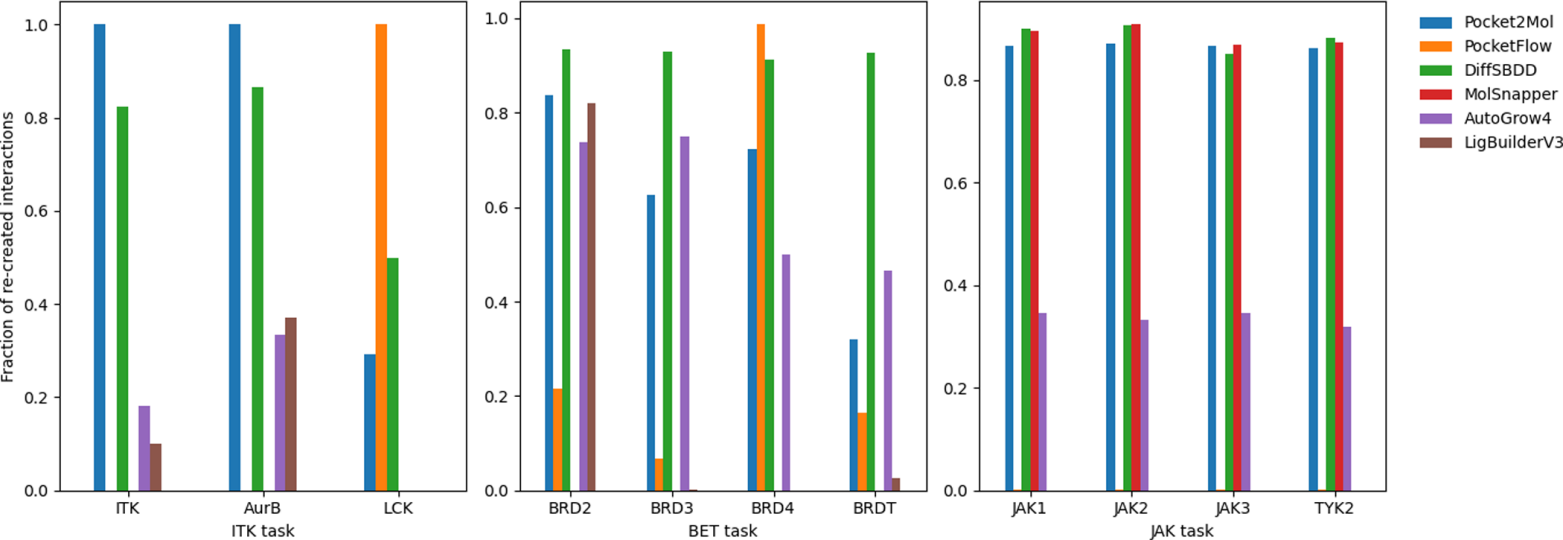
Bar charts showing the fraction of molecules from Pocket2Mol (blue), PocketFlow (orange), DiffSBDD (green), MolSnapper (red), AutoGrow4 (purple), and LigBuilderV3 (brown) with recreated active site interactions for the ITK task (left), BET task (center), and JAK task (right).

**Figure 8:**
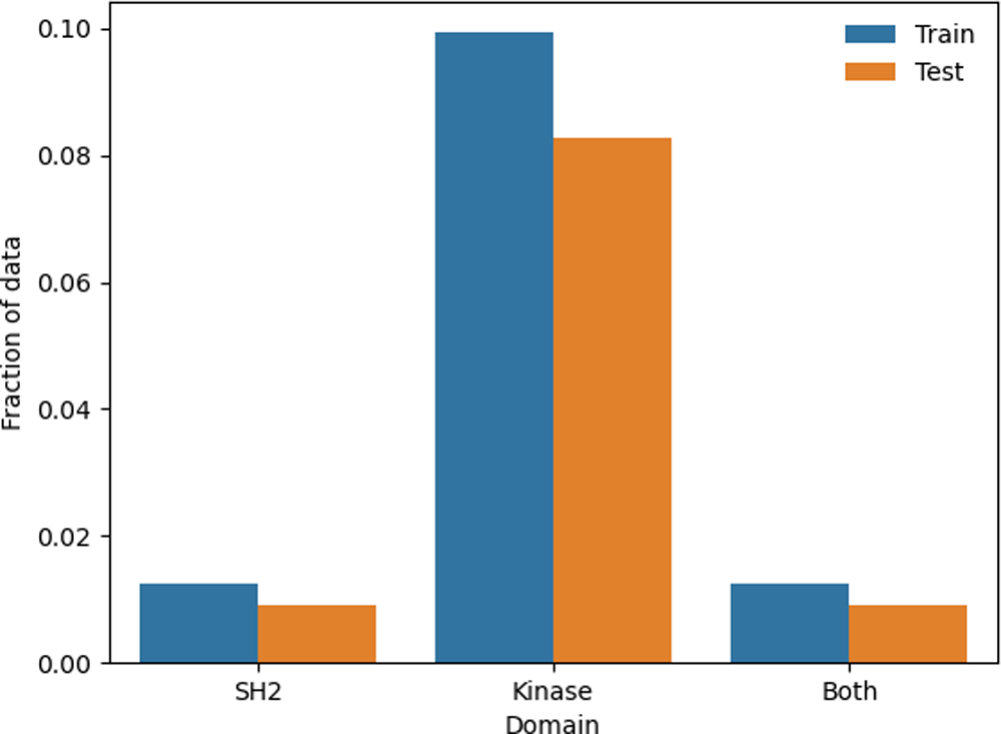
A bar chart showing the distribution of generic kinase and less frequent kinase SH2 domains in the BindingMOAD training and test sets.

Further evaluation of mean active site interactions for each task in Figure 9 and Table SI 23 showed low fractions of recreated interactions and large standard deviations for LigBuilderV3, AutoGrow4, and PocketFlow. Conversely, DiffSBDD showed large fractions of recreated interactions with smaller standard deviations. This suggests DiffSBDD, Pocket2Mol, and MolSnapper consistently recreate the most interactions required across tasks.

**Figure 9:**
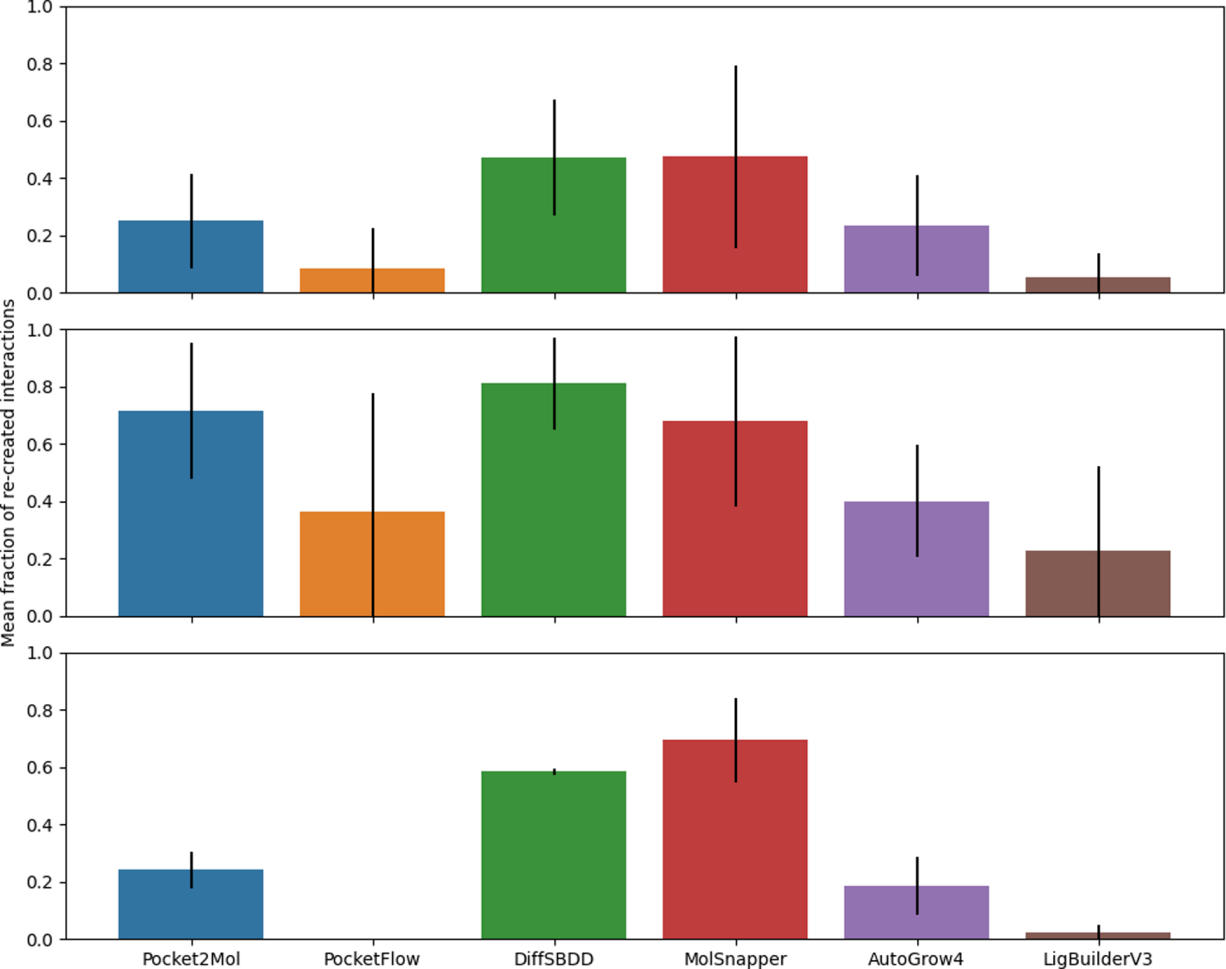
Fraction of recreated interactions with error bars displaying standard deviations across generators for task 1 (top), task 2 (middle), and task 3 (bottom).

Smith et al.^96^ highlighted that Pocket2Mol was better in interaction creation, whereas DiffSBDD was shown to focus on volume occupancy. Therefore, an analysis was performed in this study using GRID^3^ interactions generated in MOE using probe atoms to identify hydrophobic, Hydrogen-bond donor, Hydrogen-bond acceptor, and aromatic regions on the receptor with respect to the crystal ligand. Performed on ligands generated for ITK PDB 4L7S, RDKit was used to extract hydrophobic, donor, acceptor, and aromatic pharmacophores on each ligand.^81^ These were overlaid on the generated interaction points. Figure 10 illustrates the oversampling of ligand pharmacophores in DiffSBDD where volume takes precedence over interactions. This suggests the reason a higher percentage of interactions is observed in DiffSBDD over Pocket2Mol is due to the random placement of pharmacophoric points across the whole pocket. While AutoGrow4 shows similar volume occupancy to DiffSBDD, it does not oversample on any particular pharmacophore. Additionally, Pocket2Mol and MolSnapper (due to its pharmacophoric point sampling) generate ligand pharmacophore points closer to residues in the pocket rather than filling the volume. LigBuilderV3 also performs in a similar way, with a wider exploration of the protein pocket. These conclusions for DL models are consistent with those in Smith et al.^96^ Interestingly, assessing the protein–ligand interaction fingerprints extracted in MOE seen in Figure SI 4, LigBuilderV3 places greater importance on hydrogen bond interactions. This is possibly due to the SCORE empirical scoring function placing greater importance on these intermolecular interactions due to its training data.

**Figure 10:**
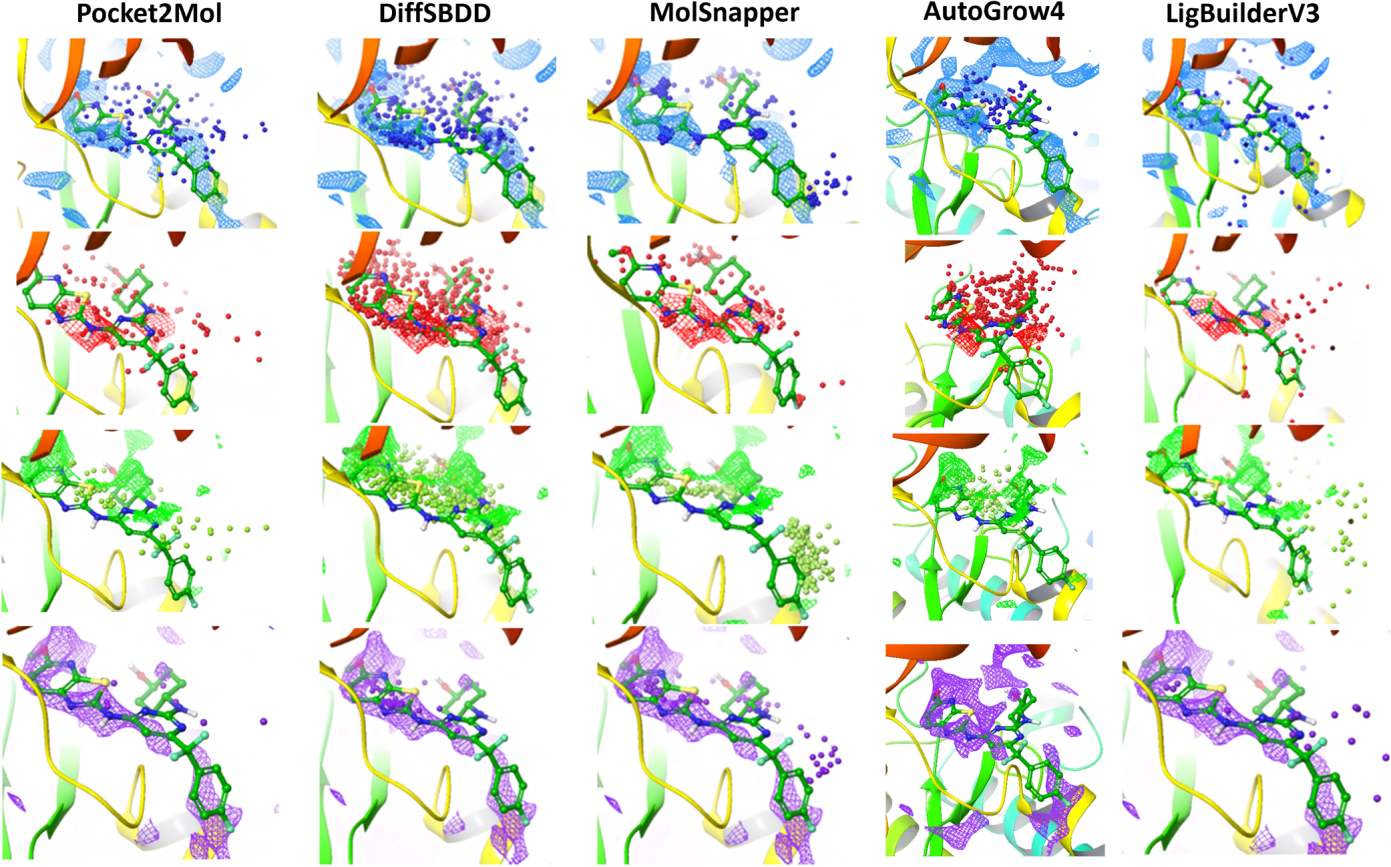
Meshed surfaces extracted from molecular interaction-derived interactions where green are hydrophobic, red are acceptors, blue are donor/acceptor regions, and purple are aromatic interactions. Each row represents ligand pharmacophore points generated in RDKit for 100 randomly sampled compounds with raw poses generated for ITK PDB 4l7s. These are empty for PocketFlow as molecules are generated far from the active site.

### Timing

The computational time needed to train and run inference on models^76^ can be seen in Tables 5 and 6. Timing and resource considerations are important in the cost assessment of traditional methods and DL models. Pocket2Mol and DiffSBDD have similar time and resource requirements for training with PocketFlow being slightly slower. During the generation process, there were many compounds that did not pass Pocket2Mol and MolSnapper’s filters, resulting in fewer than the requested compounds being generated. However, the combinatorial methods AutoGrow4 and LigBuilderV3 are much slower during inference due to the more expensive docking evaluation required for the former, the retrosynthesis for synthesizability required for the latter, and the multiple generations required for both GA methods.

**Table 5:** Retraining Time and Compute Resources Required for DiffSBDD, Pocket2Mol, and PocketFlow where Time Is in Format Days-Hours:Minutes:Seconds

	Pocket2Mol	DiffSBDD	PocketFlow
wallclock time	2–04:08:06	4–21:45:07	6–06:59:48
GPU compute resource	4	2	2

**Table 6:** Inference Time and Compute Resources Required for Each Molecule Generator to Generated 1000 Compounds where Time is in Format Days-Hours:Minutes:Seconds

	Pocket2Mol	DiffSBDD	PocketFlow	MolSnapper	AutoGrow4	LigBuilderV3
wallclock time	0–00:41:00	0–00:41:16	0–00:35:41	0–11:29:41	10–00:00:00 (max)	6–14:00:00
time per compound/s	2.5	2.5	2.1	41.4	864.0 (without limit)	568.7
compute resource	1 task, 1 CPU, 1 GPU	1 task, 1 CPU, 1 GPU	1 task, 1 CPU, 1 GPU	1 task, 1 CPU, 1 GPU	4 tasks, 1 CPU	4 tasks, 1 CPU

## Discussion

Our benchmark and analysis illustrates that 3D structure-based DL generators: (1) struggle with chemical validity, (2) struggle with generating good 3D conformations, (3) recreate more interactions within the active site compared to combinatorial methods, although DiffSBDD oversamples pharmacophores, and (4) perform faster inference compared to combinatorial methods.

### The Future of Molecule Generators

As shown by the comparison of the CrossDocked- and BindingMOAD-trained Pocket2Mol models in Figure SI 9, data set size has some impact on the chemical validity of generated compounds. To improve on this and 3D conformations, large pretrained models should be used for learning the chemistry and 3D conformational space of molecules, before being fine-tuned and used in the generator. Experimental protein–ligand crystal structures are scarce in terms of conformations and diversity of pocket types and ligand structures, where transfer learning and fine-tuning approaches could be explored further. The multifidelity approach, MFBind,^97^ has shown that pretraining can be utilized with lower-quality docking scores and then fine-tuned using physics-based calculations. Generated compounds can be sampled to have higher-quality calculations run in an active learning approach. Alternatively, optimization approaches such as Reinforcement Learning could be explored further as these have been shown to improve the chemical validity and strained conformations of compounds generated by Pocket2Mol.^98^

As most structure-based generation methods rely on protein structure to provide context for conditioning, they avoid taking protein–ligand interactions into account and can be sensitive to the amount and resolution of experimental protein structures. Training solely on protein or ligand positions has shown overfitting, whereas interaction-based methods seem more robust.^99–101^ Protein flexibility should also be evaluated with and without interaction models, as the former should theoretically be more affected by small changes in protein conformations.

Since DL methods are faster, they could be used for ideation stages where a large number of diverse compounds are required whereas combinatorial approaches could be used during later phases of drug discovery such as lead optimization. However, ideally, a molecule generator should incorporate all aspects of early stage drug discovery: hit identification, lead optimization, scaffold hopping, and linker design. Improvements have been made in this field where, similar to DiffSBDD, PNDM is a structure-based diffusion model that changes its sampling strategy to noise the required scaffolds or fragments into the active site to act as part of the protein context for the generator. These are then denoised along with the generated molecule.^102^ Different molecule generators are also complementary to one another and their combinations should be further explored, similar to Chemistry42 developed by InsilicoMedicine.^103^

Low docking scores have been shown to correspond to promiscuous compounds (showing low docking scores in other pockets), therefore, selectivity metrics should be embedded into the model.^104^ Additionally, using docking as a scoring metric in 3D generative models has been shown to overfit binding site interactions, giving less importance to 3D ligand conformation, and should be avoided for future work.^54^

Finally, throughout the generation process, the monitoring and constraining of molecules toward desired property space will be important in alleviating attrition issues of current drug discovery campaigns.

### Benchmark Further Work

Although this benchmark focuses on the recreation of important interactions for Tasks 1 and 2, it seems the generators assessed do not perform well on these tests. Certain interactions are important for the potency and selectivity of inhibitors, however, there are many ways a ligand can interact with an active site. Additionally, the same predicted interaction can vary in its strength through movement leading to changes in distance and angle, leading to different binding affinities, however, this is not currently considered in the benchmark. DiffInt was developed to explicitly model Hydrogen bond interactions through the introduction of pseudoparticles into the ligand-protein representation.^105^ A comparison with Pocket2Mol, DiffSBDD, and FLAG, showed DiffInt sampled compounds with stronger Hydrogen bonds and better represented the CrossDocked2020 training data. This was followed by DiffSBDD, however, we have shown in the current study that DiffSBDD oversamples pharmacophores (Figure 10) and does not focus on interaction recreation, which could be a similar finding for DiffInt.

The benchmark might have limitations on which chemical structures are most feasible with the proteins studied based on our knowledge of current hit compounds and binders, hence decreasing the novelty of generators and biasing benchmarking efforts toward testing for similar structures, particularly for the virtual screening task. Effects of the solvent, protein conformation, entropic contributions, and weaker van der Waals interactions are important factors for ligand binding and should be considered further to improve the benchmark. Additionally, as seen with the oversampling of pharmacophores by DiffSBDD, generators have biases toward certain groups, such as hydrogen bond acceptors, which might be greatly favored for the interaction recreations being tested. This could be improved by considering the pharmacophores of generated compounds against those of the protein active site to understand the complementarity and biases of ligands in active sites where an example score using SC_RDKit_ has been added into the analysis. 2D chemical similarity is still of importance in finding recovered active compounds for virtual screening tasks.

Finally, to assess the randomness of the generative models, two runs were performed with the exact same inputs for each of the six models evaluated in this study. It was found that the compounds generated changed depending on the run, suggesting that the best performing compounds may not have been evaluated for each model. Further analysis of seeds will be important in ensuring reproducibility of results.

## Conclusions

A benchmark was created to assess the ability for deep learning and combinatorial generators to recreate important active site interactions. An evaluation of the methods Pocket2Mol,^32^ PocketFlow,^45^ DiffSBDD,^46^ MolSnapper,^50^ AutoGrow4,^7^ and LigBuilderV3 ^8^ showed the combinatorial methods performed well when generating valid 3D ligand conformations which were difficult for deep learning models to generate. However, combinatorial methods are quite slow during inference and fail to generate sufficient numbers of compounds required for the benchmark and/or fail on 2D evaluation metrics from MOSES. Finally, the deep learning methods showed higher percentages of active site interactions recreated. After an extensive analysis was performed, a number of improvements for 3D structure-based generators are proposed to further this field such as the use of large pretrained networks, incorporation of explicit protein–ligand interactions, and use of property constraints during molecule generation.

## Supplementary Material



## Data Availability

The data and splits used in this study are available from BindingMOAD^66^ and our Apache 2.0-licensed GitHub repository (github.com/gskcheminformatics/SBDD-benchmarking), respectively. Further information on structure cleaning for the benchmark is provided in the Supporting Information and cleaned PDBs and outputs can be found on Zenodo at zenodo.org/records/15348548.

## Notes

Detailed technical descriptions of each molecule generator used in the benchmark; graph showing the comparison of common 2D metrics across published deep learning generators (2017–2023); information on PDBs used in each task with details on preparing structures and assessment strategies; distributions of training and test sets of molecular weight, synthetic accessibility score, log*P*, and tPSA; distributions of mmseqs and Enzyme Commission Numbers; Pocket2Mol, PocketFlow, DiffSBDD, MolSnapper, AutoGrow4, and LigBuilderV3 benchmark output tables; protein–ligand interaction fingerprints for each method for the ITK task; synthesizability as similarity comparisons to a vendor database; Wasserstein property distributions of log*P*, synthetic accessibility score, QED, and molecular weight; normalized spacial score distributions of each method; Pocket2Mol further retraining loss curves, allene and fused ring passes, and recreation of interactions for proteins in task 2; RMSDs of 3D conformations upon minimization for each method on PDB ID 4Q6R; and fractions of recreated interactions and standard deviations across tasks and generators (PDF)
