# Supplementary material for: Benchmarking 3D Structure-Based Molecule Generators
Source: J Chem Inf Model. 2025 Jul 25;65(15):8006–21. doi: 10.1021/acs.jcim.5c01020 (PMC12344697; doi:10.1021/acs.jcim.5c01020)
Supplement: Supplementary file 1 [file ci5c01020_si_001.pdf]

Supporting Information
Benchmarking 3D Structure-Based Molecule Generators

Natasha Sanjrani,[*,†,‡] Damien Coupry,[†] Peter Pogány,[†] David S. Palmer,[‡] and Stephen D. Pickett[†]

†*Department of Cheminformatics, Research Technologies, GSK, Gunnels Wood Road, Stevenage, SG1 2NY, UK*

‡*Department of Pure and Applied Chemistry, University of Strathclyde, Thomas Graham Building, 295 Cathedral Street, Glasgow, G1 1XL, UK*

E-mail: natasha.x.sanjrani@gsk.com

Generator Details

Pocket2Mol[1]

During training, training set atoms and bonds are masked then predicted. Negative examples are also included through sampling "nothing" atoms which are empty spaces not occupied by atoms or bonds. Protein atoms are represented through atom type, 3D coordinates, amino acid type, and backbone or side-chain atom. Ligand molecule atoms are represented through atom types, 3D coordinates, atomic valence, and the number of chemical bonds. Finally, bonds are represented through bond distances, bond types, and valencies with an additional vector describing the direction of the bond.

Encoder: The protein atoms, ligand atoms, and bonds are all encoded into a k-nearest neighbour graph and an embedding is generated through the first section of the NN.

Frontier atoms: The next section of the network takes these embeddings and predicts whether each atom is a frontier, which is an atom that can connect to new atoms. If no ligand atoms have been generated thus far, this prediction is performed on protein atoms.

Position atoms: A focal atom is sampled from the frontier set of atoms, and the third part of the network generates new coordinates relative to this focal atom. This is predicted using a Gaussian Mixture Model, where the mean, covariance, and class weightings for each possible atom are predicted by separate layers in the network.

Atom and bond type: Elements are predicted through another network by collecting the nearest neighbours of the newly generated atoms and message passing to another layer to predict the probability of each atom type. Bonds are predicted similarly through collecting nearest neighbours, message passing for embedded representations of atoms i and j, to then use a layer for the prediction of the probability of a certain bond type between i and j. A self-attention mechanism is used here to ensure bond types are reasonable within their local environment e.g. two triple bonds should not be close to each other.

Each part of the network is updated according to the losses seen in equation 1, where the frontier is a binary cross-entropy term, the position is the negative log-likelihood of atom positions, and element and bond types are cross-entropy terms.

$$L = L_{frontier} + L_{position} + L_{element} + L_{bond} \tag{1}$$

PocketFlow[2]

Similar to Pocket2Mol, during training, training set atoms and bonds are masked then predicted. Protein atoms are represented through atom type, 3D coordinates, amino acid type, and backbone or side-chain atom. Ligand molecule atoms are represented through atom types, 3D coordinates, number of neighbours, atomic valence, and the number of chemical

bonds. Finally, bonds are represented through bond distances and bond types.

Context Encoder: Equivariant graph attention network used to encode the protein and molecule.

Focal Net: The next section of the network takes these context embeddings and predicts whether each atom is a frontier, which is an atom that can connect to new atoms. If no ligand atoms have been generated thus far, this prediction is performed on protein atoms. Frontier atoms that are fully saturated are discarded.

Atom Flow: After sampling a random variable from a normal distribution, this module predicts the distribution of the new atom type given the features of the focal atom.

Position Predictor: The relative position of the new atom is predicted using the focal atom and the new atom type. The distance between the new atom and focal atom is restricted to be below 2Å.

Bond Flow: Atoms within a 4Å cut-off of the newly generated atom are considered for bond formation. The module samples a random variable from the normal distribution and calculates the bond type distribution taking into account the context, new atom type, and new atom coordinates. Importantly, the following knowledge-based constraints are used here: if the bond exceeds the valence of the atom another bond is predicted, if O-O, O-N, C=C=C and 3-membered rings are formed the bond and atom are deleted and resampled, and if 6-membered rings with N or C contain 2 double bonds they are converted to aromatic rings.

Each part of the network is updated according to the losses seen in equation 2, where the focal loss is a binary cross-entropy term, the position, atom, and bond type losses are negative log-likelihoods of atom positions, elements, and bonds.

$$L = L_{focal} + L_{position} + L_{atom} + L_{bond} \tag{2}$$

DiffSBDD[3]

Encoding: Ligand and protein atoms are represented using a point cloud containing 3D coordinates and atom types. These are first passed through separate multi-layer perceptrons and then through a joint embedding layer to create a joint latent space. However, no explicit bonding information is taken into account.

Model: An SE(3)-equivariant GNN (EGNN) is used, taking the joint embedding as input, and updating representations through multiple message-passing layers. SE(3)-equivariance was used to encode compound chirality and was shown to distinguish well between chiral and non-chiral compounds. The EGNN was trained to predict the noise added to the point cloud embeddings by a pre-defined polynomial noise scheduler, clipped between 0.001 and 1. The loss is shown in equation 3 where $\hat{\epsilon}$ is the noise predicted by the EGNN. To reconstruct molecules, the noised input representation, which has noise added to it through a scheduler, has the EGNN-predicted noise taken away from it to generate a point cloud node containing atom type and 3D coordinates. This is used to train the model. It should be noted that before sampling point cloud nodes, the number of ligand atoms are sampled from the training dataset distribution, however, the authors increased this by 5 or 10 to improve docking scores.

$$L_{train} = \frac{1}{2}||\epsilon - \hat{\epsilon}||^2 \tag{3}$$

Bond prediction: OpenBabel is used to infer bonds and bond types for sampled atoms. The method uses atom proximity to add bonds if their atomic covalent radii meet a threshold, but these atoms cannot be closer than 0.4 Å. These bonds are then filtered using valence constraints.[4]

MolSnapper[5]

MolSnapper is based on the earlier diffusion model, MolDiff[6] and did not require re-training for this evaluation.

MolDiff defines a 3D molecule through its atom type, coordinates, and bond type. A forward noising process samples from a standard normal distribution to add to atomic positions and samples from categorical probability mass functions to add to atom types and bond types. E(3)-equivariant GNNs are used to predict the noise that was added to each of these processes, with a final loss function seen in equation 4 where the λ are defined hyper-parameters and the position loss is the mean-squared error between the atom position and mean sampled from the network. The atom and bond losses are Kullback-Leibler divergences between the actual and predicted probabilities of atoms and bonds. Edge information was also added into the generator, unlike that of DiffSBDD, as it was shown to be important for *de novo* graph-based generation.[7] Importantly, MolDiff defines two stages of the noise schedule where in stage 1, bonds have more noise added to them than atoms, as adding too much noise to atoms would lead to unreasonable bonds. In stage 2, noise is only added to atoms for learning. Bond predictor guidance is also added into the atom position generation process as atomic positions generated too far from one another lead to bond predictions outside of relevant ranges. Therefore, the gradient of a bond prediction confidence model is used to guide the sampling of atom positions.

$$L^{t-1} = L^{t-1}_{pos} + \lambda_1 L^{t-1}_{atom} + \lambda_2 L^{t-1}_{bond} \tag{4}$$

In MolSnapper, a 3D molecule is represented through its atom type, coordinates, and bond type. Additional features introduced by MolSnapper were pharmacophore positions and types, extracted from the original crystal ligand using RDKit, and protein atom positions. The unconstrained diffusion process was altered using masks to facilitate the generation near pharmacophore points during the reverse diffusion (generation) process. Clash

guidance was additionally incorporated into the loss function to avoid the distance between protein atoms and sampled ligand atoms becoming too close.

AutoGrow4[8]

Initial generation: The starting population is a set of diverse molecular fragments extracted from the ZINC15 drug-like chemical space. The next generations are created from preceding generations using the operators outlined below in parallel.

Elitism operator: Ranks the current population based on the fitness function and selects the highest-ranking compounds.

Mutation operator: Uses SMARTS-defined reactions in the program to mutate compounds. If additional fragments are required they are sampled from the ZINC15 library.

Crossover operator: Finds the largest substructure between two parent compounds and randomly selects other parts of their structures to generate a new child.

Filtration and fitness: After each of the operators is applied to the current generation of molecules, they are filtered on Lipinski and other defined filters such as PAINS.[9] 3D conformers are generated using Gypsum-DL[10] from the SMILES representations created using the operators above. Compounds are evaluated on a docking score, the default being Vina[11] docking, and a diversity score where fingerprint similarities are used to keep only the most diverse compounds in the current generation.

Compound ranking and selection: Different types of ranking and selection methods are employed. The base ranking method keeps only the best scoring solutions. The roulette method randomly selects solutions with a defined weighting scheme. The tournament method randomly selects a sub-population and selects the fittest from this sub-population. Each of these ranking and selection methods are applied in conjunction to the compounds in each generation.

LigBuilderV3[12]

Cavity: An algorithm, Cavity1.0, developed by the same group, uses a probe sphere to explore the surface of the protein to extract binding pockets. This has an accuracy score of approximately 86%.[13] Upon detection of a cavity, the program identifies key interaction sites using a positive nitrogen probe (hydrogen bond donor), a negative oxygen probe (hydrogen bond acceptor), and a carbon atom (hydrophobic group). The probe atoms and residues at each point have an empirical binding energy score calculated to determine donor, acceptor, and hydrophobic regions on the pocket.

The program uses a sequential building approach and was initially developed for lead optimization on provided fragments, however, this functionality is not explored further in this evaluation.[14]

Build: The *de novo* design method, Drug Space Exploration Algorithm (DSEA), was developed to generate compounds. An sp^3 Carbon atom is used as a starting point for the construction of ligands. These ligands are used as initial fragments for subsequent generations. A library of building blocks is defined by LigBuilder through the World Drug Index which is sampled and attached to a selected hydrogen atom on the input ligand/starting atom. All possible free hydrogen atoms are sampled, the molecules generated, and child compounds evaluated. The newly formed single bond is then systematically rotated to determine the best 3D conformation of the molecule. Information on bond lengths, bond angles, and van der Waals radii are taken from the Tripos forcefield to ensure valid 3D conformations.

Mutation operators are used to introduce randomness and novelty into generated structures by replacing C, N, and O atoms with one another if they are better suited to donor, acceptor, or hydrophobic regions on the defined pocket. Similar to AutoGrow4, an elitism operator is also used to keep the top-scoring compounds.

Evaluation: Generated compounds are evaluated on how well they fit into the cavity based on collisions. They are also evaluated using the SCORE empirical scoring function

that predicts binding affinity given intermolecular interactions. Molecular properties are also evaluated on generated compounds, ensuring molecules sit within certain logP and molecular weight ranges. Finally, synthesizability is assessed using a retrosynthesis algorithm where generated compounds are broken down into simple fragments, and a reagent library from ZINC8 is searched to assess whether a synthetic route can be found. Based on these rankings, a tournament selector and a roulette selector are used to progress compounds to the next generation.

Architecture Metric Comparisons

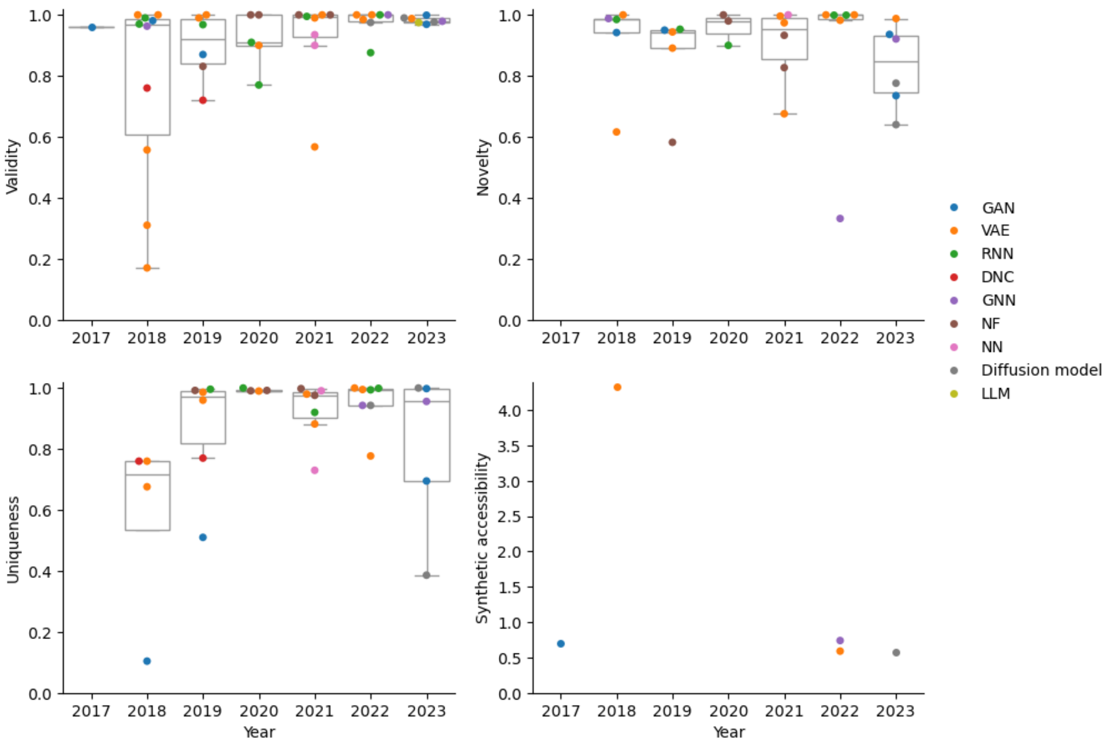

Figure SI 1: Validity, novelty, uniqueness, and synthetic accessibility metrics reported for molecule generators from 2017, coloured by model type

Task Information

Table SI 1: Table of task 1 PDB proteins selected from BindingMOAD with their assessment information. All proteins were prepared in the Schrodinger Maestro suite and t-tests were used to assess for significance between differences of distance and similarity

**Blind sample ID**	**PDB ID/s**	**Description**	**Assessment**	**Comments**	**Ref**
1	6ct5	Loss of 8918 functionality observed for the W170S mutant	Assess distance to wild-type and mutated inhibitors given wild-type and mutated structures	Ions and solvents removed, only chain A kept. Mutation re-created for the prepared protein using the mutate functionality on which energy minimization was performed +-1 amino acid.	[15]

2	1w6k; 1w6j	Complexed with natural inhibitor lonasterol	Assess affects of protein conformation by checking minimum distance to important active site interactions re-created with Asp455, Tyr237, Cys233, and Ile524		[16]

3	5cqh; 5cqi	Nucleotide bound structure to simulate the cytidine to uridine transformation; glycerol bound structure	Similarity to crystal structure and more than 50% of the time hydrophobic and hydrogen bond active site interactions are re-created	Glycerols removed as they are not in the active site, the protein is used to assess affects of changing protein conformation	[17]

4	3rde	Iron is essential for catalysis of bound inhibitor	Assess whether protein input with and without Fe affects generated molecule distance and more than 50% of the time hydrophobic and hydrogen bond active site interactions are re-created	Chain A kept	[18]

5	3hii; 3hi7; 3hig	With inhibitor pentamidine; apo structure; with inhibitor berenil	Whether more than 50% of the time hydrophobic and hydrogen bond active site interactions are re-created	Chain A kept	[19]
6	6ryp; 6ryo	Inhibitors bind in opposite directions	Whether affects of input protein conformation changes distance of generated compounds to active site	Structures aligned	[20]

7	4l9i; 4mk0	With selective paroxetine inhibitor in GRK1; with paroxetine derivative, for GRK2	Whether less than 50% of the time clashes are present and more than 50% of the time hydrogen bond active site interactions are re-created	Chain A kept	[21]
8	4q6r	With inhibitor from study	Whether more than 50% of the time hydrophobic and hydrogen bond active site interactions are re-created	Chain A kept	[22]

9	1pl6; 1pl7	Inhibitor complex with Zn, NADH, and SDH; apo structure with residues coordinating with Zn	If, for both 1pl6 and 1pl7, more than 50% of the time hydrophobic and hydrogen bond active site interactions are re-created regardless of cofactors	Chain A kept	[23]

10	6r7d	With inhibitor from study	Whether more than 50% of the time hydrophobic, hydrogen bond, and saltbridge active site interactions are re-created	Chain A kept	

11	1itu; 1itq	Complexed with cilastatin; apo form	Zn is important in binding, assess whether distance to Zn changes on protein structure with and without Zn for apo and holo forms and if more than 50% of the time hydrophobic, hydrogen bond, and saltbridge interactions are re-created	Chain A kept, structures aligned	[24]

12	2v77	With strongly bound inhibitor	Whether more than 50% of the time hydrophobic and hydrogen bond interactions are re-created with and without Zn in input and whether distance of generated compound to Zn changes	Chain A kept	[25]

13	2c97; 2c9b; 2c92; 2c94; 2c9d	Chloro pyrimidine inhibitor; the rest are with a purinetrione inhibitor	Whether, for each protein conformation, more than 50% of the time hydrophobic and hydrogen bond active site interactions are re-created	Chain A kept, structures aligned	
14	5jwc; 5jwa	Two binding modes exhibited	Whether both binding modes are found	Structures aligned	[26]

15	6z80; 6z85	Stimulatory with 8-oxo bound in active site near region B; Inhibitory with BH4 bound in allosteric site near region A	Whether more than 50% of the time hydrogen bond active site interactions are re-created	Chain A kept; chain J kept	[27]

16	5yjw; 5yjx; 5yjy	With stigmatellin; with AC0-12; with myxothiazol	Contains active and allosteric sites, assess whether both of these pockets are found and if generated compound is less than 5 Angstroms to crystal and if more than 50% of the time hydrophobic and hydrogen bond interactions are re-created	Chain A and FAD cofactor kept	[28]

17	6xi9; 6xig	With inhibitor; apo form	Whether, for each protein conformation with and without SF4, more than 50% of the time hydrophobic, hydrogen bond, and saltbridge active site interactions are re-created	Chain A and SF4 kept, Cl removed	[29]

18	1kqb; 1kqc; 1kqd	With benzoate inhibitor; with acetate inhibitor; reduced form where flavin mononucleotide bends	Whether more than 50% of the time hydrophobic and hydrogen bond active site interactions are re-created	Chains A and B kept	[30]
19	4kxb; 4kxc; 4kxd	With bestatin inhibitor; glutamate in active site; glutamate and Ca in active site	H2O kept and removed as it is important in complexing with Ca, assess whether more acidic groups are generated on compounds with Ca in active site	Zn kept	

20	5iki; 4yt3	With abietic acid; with acetate	Whether, for both apo and holo forms, more than 50% of the time hydrophobic and hydrogen bond active site interactions are re-created	Chain B kept and structures aligned	[31]

Table SI 2: Table of task 2 PDB proteins with their assessment information. All proteins were prepared in the Schrodinger Maestro suite

**Task subset**	**Protein**	**PDB ID**	**Comments**	**Removed from training**	**Assessment**

Selectivity ITK/LCK and ITK/AurB	ITK	4l7s		Y	Whether more than 50% of the time hydrogen bond active site interactions are re-created and distance to Val419 changes given ITK or AurB
	LCK	1fbz	Only chain A kept	Y	
	AurB	4af3	Only chain A kept	Y	

Pan-JAK inhibitors	JAK1	5wo4	Only chain A kept	Y	Whether more than 50% of the time hydrogen bond active site interactions are re-created
	JAK2	7q7k		Not in BindingMOAD	
	JAK3	7q6h	PHU ligand kept in complex	Not in BindingMOAD	
	TYK2	3lxn		Y	

Pan-BET activity	BRD2 domain 1	6ddi	Only chain A kept		Whether more than 50% of the time hydrogen bond active site interactions are re-created and compounds are less than 5 Angstroms from the WPF motif and ZA channel
	BRD2 domain 2	5ig6			
	BRD3 domain 1	7lay	Only chain A kept		
	BRD3 domain 2	3s92			
	BRD4 domain 1	4qb3			

	BRD4 domain 2	7usg			
	BRDT domain 1	7mrd			
	BRDT domain 2	7l99	Only chain A kept		
Shikimate kinase		1zyu		Not in BindingMOAD	Whether more than 50% of the time the Arg58 and Asp32 interactions are re-created

Table SI 3: Table of task 3 PDB proteins with their assessment information. All proteins were prepared in the Schrodinger Maestro suite

**Task subset**	**Protein**	**PDB ID**	**Additional information**	**Assessment**

COVID Moonshot	Main Protease	7gax	A lot of PDBs available, best PDB score chosen	Whether more than 50% of the time hydrogen bond active site interactions are re-created and the average 2D similarity is >0.5
CSAR 2014	FXa	4zh8, 4y6d, 4zha, 4y71, 4y76, 4y79, 4y7a, 4y7b		Whether more than 50% of the time hydrogen bond active site interactions are re-created and the average 2D similarity is >0.5
	SYK	4yjo, 4yjp, 4yjq, 4yjr, 4yjs, 4yjt, 4yju, 4yjv	Structures were aligned	

	TrMD	4ypw, 4ypx, 4ypy, 4ypz, 4yq0, 4yq1, 4yq2, 4yq3, 4yq4, 4yq5, 4yq6, 4yq7, 4yq8, 4yq9, 4yqa, 4yqb, 4yqc, 4yqd, 4yqg, 4yqi, 4yqj, 4yqk, 4yql, 5d9f, 4yqn, 4yqo, 4yqp, 4yqq, 4yqr, 4yqs, 4yqt	Structures were aligned	

Table SI 4: SMARTS patterns for filters used in the structure-based benchmark

**Filter**	**SMARTS**
**Allene**	[$([C;R]=[C]=[C]),$([C]=[C;R]=[C])]=[C]=[C]
**Fused ring**	*~&@*(~&@*~&@*(~&@*)~&@*~&@*)~&@*~&@*(~&@*)~&@*~&@*
	(~&@)~&@*(~&@*(~&@*~&@*)~&@*(~&@*)~&@*(~&@*~&@*)~&@*(~&@*~&@*)~&@*)~&@*~&@*

	(~&@)(~&@*(~&@*(~&@*(~&@*)~&@*)~&@*(~&@*)~&@*)~&@*(-&@*)~&@*)~&@*
**MOSES MCF**	moses/metrics/mcf.csv
**MOSES PAINs**	moses/metrics/wehi_pains.csv

Dataset analysis

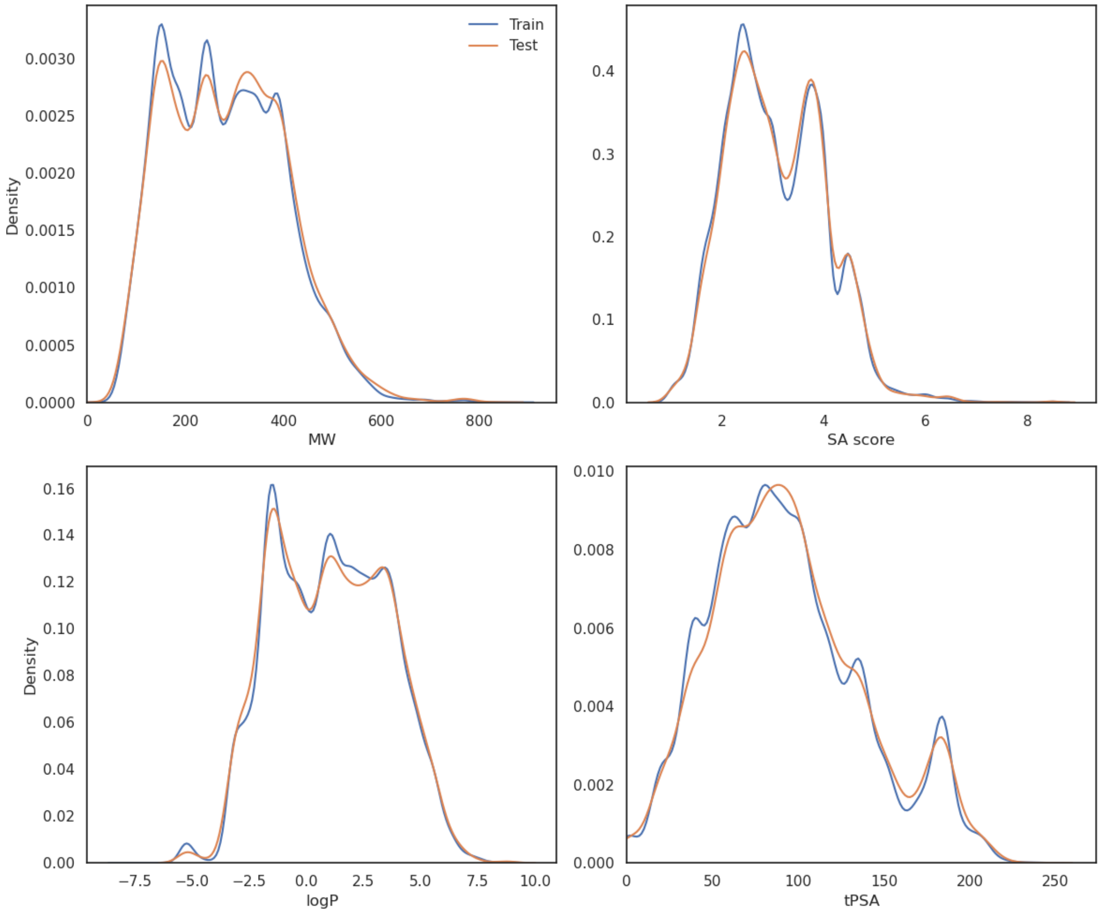

Figure SI 2: Distributions of training and test sets of molecular weight, synthetic accessibility (SA) score, logP, and tPSA.

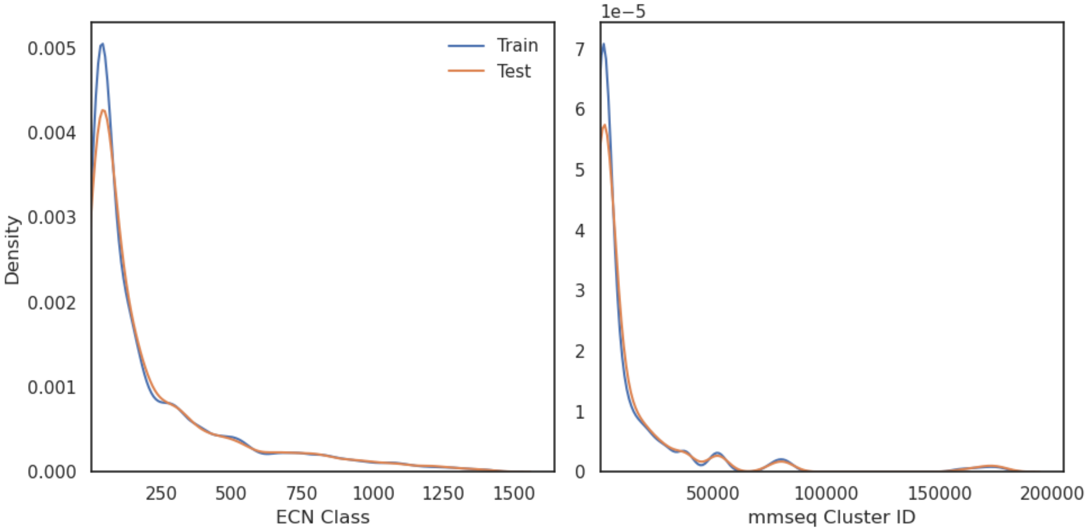

Figure SI 3: Distributions of cluster IDs for enzyme commission numbers (ECNs) and mmseqs2 for the BindingMOAD dataset. The ECNs were renumbered to aid in the visualization of the density plot.

Pocket2Mol outputs

Table SI 5: Table of task 1 Pocket2Mol full outputs for each protein, with reasons given for failures and percentage of passes in each category

prot	comments	passed	molgen	MOSES	bond__length	bond__angle	int__clash	int__energy	dist__to__prot	dist__to__wat	vol__prot
**1**		TRUE	69.01	86.6	94.86	99.73	99.19	97.56	60.49	100	89.45
**2**		Generation and/or analysis not run;									
**3**	Step3/4: Did not pass hydrophobic re-creation check;	FALSE	53.79	80.34	94.64	99.54	98.39	94.72	27.93	100	98.01
**4**	Step2/4: Did not pass t-test with Fe and without Fe;	FALSE	23.96	86.04	92.64	96.6	100	92.64	39.43	100	87.92
**5**	Step2/3: Did not pass hydrophobic re-creation check;	FALSE	44.59	74.69	79.12	99.88	98.53	96.19	31.94	99.63	85.14
**6**	Step2/3: Did not pass 6ryo distance t-test;	FALSE	44.6	87.41	94.6	98.2	97.48	93.53	69.42	100	100
**7**	Step1/3: Did not have sufficient data points;	FALSE	48.94	63.83	92.2	99.65	98.58	78.37	67.73	99.65	98.94
**8**	Step2/3: Did not pass hbond re-creation check;	FALSE	70.85	73.37	97.74	100	97.74	93.22	75.38	100	100

**9**	Step2/4: Did not pass 1pl6 H2O and no H2O hbond and hydrophobic interactions check;Did not contain sufficient 1pl7 data;	FALSE	40.08	75.79	89.93	99.15	96.46	94.17	58.18	85.31	99.62
**10**	Step2/4: Did not pass hbond re-creation check;	FALSE	53.85	88.03	95.3	100	99.15	96.15	50	100	97.86
**11**	Step4/5: Did not pass apo hydrophobic, hbond, saltbridge re-creation checks;	FALSE	55.3	73.97	83.13	98.38	99.48	92.06	24.35	100	88.75
**12**	Step2/4: Did not pass hydrohobic, hbond, saltbridge re-creations with Zn check;	FALSE	65.37	69.37	93.9	99.22	98.78	94.89	36.29	100	99.33
**13**	Step1/6: Did not contain sufficient data points;	FALSE	53.73	86.78	91.71	99.4	99.4	91.47	84.38	99.76	99.76
**14**		TRUE	44.9	40.71	52.87	57.69	57.39	53.61	35.68	57.95	57

**15**	Step2/2: Did not pass hbond re-creation check;	FALSE	25.99	85.99	88.3	98.23	95.51	92.52	100	100	100
**16**	Step3/4: Did not pass hbond and hydrophobic re-creations check;	FALSE	14.82	82.55	94.42	99.44	98.41	94.18	64.78	97.93	96.25
**17**	Step1/7: Did not pass apo with and without SF4 hydrophobic re-creation check;Step3/7: Did not pass apo with and without SF4 hbond re-creation check;Step6/7: Did not pass apo with and without SF4 saltbridge re-creation check;	FALSE	26.62	84.44	89.79	97.72	99	92.43	49.04	99.86	99.5
**18**	Step2/3: Did not pass hydrophobic re-creation check;	FALSE	39.98	78.73	96.27	99.07	99.61	93.17	100	100	100
**19**		TRUE	36.74	75.68	92.14	99.49	99.45	93.33	100	100	100

**20**	Step1/3: Did not contain sufficient data points; Step2/3: Did not pass apo hydrophobic and hbond re-creation check;	FALSE	19.69	93.7	92.91	99.21	96.06	92.91	70.08	85.04	98.43

Table SI 6: Table of task 2 Pocket2Mol full outputs for each protein, with reasons given for failures and percentage of passes in each category

prot	comments	passed	molgen	MOSES	bond__length	bond__angle	int__clash	int__energy	dist__to__prot	dist__to__wat	vol__prot
**ITK**	Step2/3: Did not pass hbond ITK, LCK, AurB re-creation check;	FALSE	47.61	79.26	97.87	99.73	98.67	92.29	65.43	100	99.47
**JAK**		TRUE	31.46	86.78	74.62	98.36	98.25	93.68	66.2	100	99.53
**BET**	Step2/4: Did not pass hbond BRD2, BRD3, BRD4, BRDT re-creation check;	FALSE	31.29	86.7	92.14	99.62	98.17	94.29	80.34	100	99.89
**shik**	Step2/3: Did not pass Asp32 hbond re-creation check;	FALSE	59	75.81	95.58	95.28	99.41	94.1	68.14	95.87	100

Table SI 7: Table of task 3 Pocket2Mol full outputs for each protein, with reasons given for failures and percentage of passes in each category

**prot**	**comments**	**passed**	**molgen**	**MOSES**	**bond__length**	**bond__angle**	**int__clash**	**int__energy**	**dist__to__prot**	**dist__to__wat**	**vol__prot**
**Moonshot**		TRUE	37.5	61.88	92.5	99.38	100	92.81	66.25	100	99.06
**CSAR**		TRUE	44.23	82.43	93.2	97.62	98.43	91.95	54.26	99.98	97.77

PocketFlow outputs

Table SI 8: Table of task 1 PocketFlow full outputs for each protein, with reasons given for failures and percentage of passes in each category

**prot**	**comments**	**passed**	**molgen**	**MOSES**	**bond__length**	**bond__angle**	**int__clash**	**int__energy**	**dist__to__prot**	**dist__to__wat**	**vol__prot**
**1**		TRUE	99.60	95.80	95.80	96.20	83.60	18.40	37.40	100.00	88.40
**2**	Analysis not run or less than 100 compounds generated;										
**3**	Step3/4: Did not pass hydrophobic re-creation check;	FALSE	99.90	44.30	91.00	93.80	66.40	50.60	18.00	100.00	29.50
**4**	Step2/4: Did not pass t-test with Fe and without Fe;	FALSE	100.00	54.80	96.80	99.60	90.00	54.40	33.60	100.00	17.60
**5**	Step2/3: Did not pass hydrophobic re-creation check;	FALSE	99.87	30.40	90.00	92.87	71.73	52.33	7.47	99.80	14.53
**6**	Step2/3: Did not pass 6ryo distance t-test;	FALSE	99.80	83.00	94.90	96.50	82.30	46.00	50.90	100.00	87.10
**7**	Step2/3: Did not pass hbond re-creation check;	FALSE	99.00	87.90	95.70	97.20	87.50	25.80	79.80	100.00	99.80

**8**	Step2/3: Did not pass hbond re-creation check;	FALSE	99.80	62.60	80.80	89.00	71.40	57.80	0.00	99.20	0.00
**9**	Step2/4: Did not pass 1pl6 H2O and no H2O hbond and hydrophobic interactions check;Did not contain sufficient 1pl7 data;	FALSE	99.90	72.00	93.60	95.10	75.70	41.20	14.30	99.80	0.00
**10**	Step3/4: Did not pass hydrophobic re-creation check;	FALSE	100.00	93.40	74.20	98.60	90.00	61.40	43.60	100.00	97.40
**11**	Step2/5: Did not pass apo Zn no Zn distance check;	FALSE	98.10	75.00	88.30	93.70	80.50	53.70	63.20	100.00	97.80
**12**	Step2/4: Did not pass hydrohobic, hbond, saltbridge re-creations with Zn check;	FALSE	99.80	55.00	92.80	96.00	67.20	59.40	8.60	100.00	0.20
**13**	Step1/6: Did not contain sufficient data points;	FALSE	100.00	65.68	90.96	96.84	77.00	36.40	16.04	99.96	37.00

**14**	Step2/2: Did not pass 2 clusters check for binding sites;	FALSE	100.00	91.10	98.10	98.20	91.10	81.40	3.50	100.00	0.00
**15**	Step2/2: Did not pass hbond re-creation check;	FALSE	99.90	83.90	95.80	97.90	88.60	49.90	85.40	100.00	97.40
**16**	Step2/4: Did not pass active and allosteric pocket finding checks;	FALSE	99.80	57.07	96.73	97.53	76.27	61.93	38.13	100.00	10.00

**17**	Step1/7: Did not pass apo with and without SF4 hydrophobic re-creation check; Step3/7: Did not pass apo with and without SF4 hbond re-creation check;Step6/7: Did not pass apo with and without SF4 saltbridge re-creation check;	FALSE	99.90	54.90	67.40	98.10	64.50	75.60	14.00	100.00	0.00
**18**	Step2/3: Did not pass hydrophobic re-creation check;	FALSE	99.80	59.27	81.60	95.20	80.00	31.00	0.00	0.00	0.00
**19**	Step2/2: Did not pass having more acidic groups with Ca;	FALSE	99.73	68.53	93.47	94.73	73.53	35.27	22.93	87.20	29.20

**20**	Step2/3: Did not pass apo hydrophobic and hbond re-creation check;	FALSE	99.90	75.30	93.70	97.20	84.80	36.20	12.30	88.70	25.10

Table SI 9: Table of task 2 PocketFlow full outputs for each protein, with reasons given for failures and percentage of passes in each category

prot	comments	passed	molgen	MOSES	bond__length	bond__angle	int__clash	int__energy	dist__to__prot	dist__to__wat	vol__prot
**ITK**	Step2/3: Did not pass hbond ITK, LCK, AurB re-creation check;	FALSE	99.93	84.53	86.03	95.5	85.37	63.93	29.30	100.00	31.40
**JAK**	Step3/2: Did not pass hbond JAK1, JAK2, JAK3, TYK2 re-creation check;	FALSE	99.98	67.58	92.05	95.25	75.12	43.95	40.80	99.98	31.62
**BET**	Step2/4: Did not pass hbond BRD2, BRD3, BRD4, BRDT re-creation check;	FALSE	99.77	79.09	92.20	96.50	85.87	44.07	61.76	100.00	93.17
**shik**		TRUE	99.60	90.50	91.90	96.60	88.00	47.80	64.70	67.70	80.70

Table SI 10: Table of task 3 PocketFlow full outputs for each protein, with reasons given for failures and percentage of passes in each category

**prot**	**comments**	**passed**	**molgen**	**MOSES**	**bond__length**	**bond__angle**	**int__clash**	**int__energy**	**dist__to__prot**	**dist__to__wat**	**vol__prot**
**Moonshot**	Step2/3: Did not pass hbond re-creation check;	FALSE	99.70	94.30	95.90	96.50	88.90	47.30	76.30	100.00	96.40
**CSAR**	Analysis not run or less than 100 compounds generated;										

DiffSBDD outputs

Table SI 11: Table of task 1 DiffSBDD full outputs for each protein, with reasons given for failures and percentage of passes in each category

prot	comments	passed	molgen	MOSES	bond__length	bond__angle	int__clash	int__energy	dist__to__prot	dist__to__wat	vol__prot
**1**		TRUE	99.6	56.3	75	71.4	77.5	76	30.4	100	55
**2**	Analysis not run or less than 100 compounds generated;										
**3**	Step1/4: Did not have sufficient data points;	FALSE	99.3	58.4	60	61.8	51.5	68	0	100	0.2
**4**	Step2/4: Did not pass t-test with Fe and without Fe;	FALSE	97.3	40.4	86.9	78.5	79	82.2	47.1	100	99.1
**5**	Analysis not run or less than 100 compounds generated;										
**6**	Step2/3: Did not pass 6ryo distance t-test;	FALSE	96.79	45.15	94.04	77.28	86.16	81.02	56.95	100	99.36
**7**	Step1/3: Did not have sufficient data points;	FALSE	90.5	43.4	93.8	82.5	85.1	80.8	83.1	100	100
**8**	Step3/3: Did not pass hydrophobic re-creation check;	FALSE	99.8	68.1	97	91.5	93.8	89.5	93.1	100	99.7

**9**	Step2/4: Did not pass 1pl6 H2O and no H2O hbond and hydrophobic interactions check;Did not contain sufficient 1pl7 data;	FALSE	97.25	65	95.35	88.4	91.55	87.75	80.45	98.1	99.55
**10**	Generation and/or analysis not run;										
**11**	Step4/5: Did not pass apo hydrophobic, hbond, saltbridge re-creation checks;	FALSE	98.65	58.2	94.2	86.45	89.05	0	80.3	100	99.65
**12**	Step2/4: Did not pass hydrohobic, hbond, saltbridge re-creations with Zn check;	FALSE	99.4	56.6	90.9	79.4	85.3	83.6	51.2	100	96.9
**13**	Step1/6: Did not contain sufficient data points;	FALSE	98.28	59.77	94.77	91.15	89.92	87.58	83.58	99.85	94.8
**14**		TRUE	97.85	58.4	94.25	85.65	88.5	83.85	74.05	100	96.8

**15**	Step2/2: Did not pass hbond re-creation check;	FALSE	99.45	62.3	91	86.6	87.7	0	36.95	100	67.9
**16**	Step3/4: Did not pass hbond and hydrophobic re-creations check;	FALSE	93.7	47.15	92.4	81.45	85.45	0	48.4	96	83.45
**17**	Step5/7: Did not contain sufficient data points;	FALSE	97.35	43.4	90.9	78.75	84.35	81.25	41.05	99.65	98.7
**18**	Step1/3: Did not contain sufficient data points; Step2/3: Did not pass hydrophobic re-creation check;	FALSE	98.9	58.95	92.95	89.8	87.5	86	36.4	82.15	60.9
**19**		TRUE	96.47	41.7	90.6	78.93	81.37	79.63	62.57	95.63	98.17
**20**	Step1/3: Did not contain sufficient data points; Step2/3: Did not pass apo hydrophobic and hbond re-creation check;	FALSE	97.5	48.3	91.4	83	86.2	81.9	90	97.7	100

Table SI 12: Table of task 2 DiffSBDD full outputs for each protein, with reasons given for failures and percentage of passes in each category

**prot**	**comments**	**passed**	**molgen**	**MOSES**	**bond__length**	**bond__angle**	**int__clash**	**int__energy**	**dist__to__prot**	**dist__to__wat**	**vol__prot**
**ITK**	Step2/3: Did not pass hbond ITK, LCK, AurB re-creation check;	FALSE	94.27	48.03	79.67	73.07	73.03	74.27	55.33	100	66.47
**JAK**		TRUE	86.88	45.82	90.5	83.15	83.85	79.35	82.12	100	99.85
**BET**	Step3/4: Did not create compounds <5 A from WPF motif	FALSE	96.42	63.7	95.05	90.77	92.27	0	86.42	100	99.8
**shik**	Step2/3: Did not pass Asp32 hbond re-creation check;	FALSE	97.7	57.2	93.3	81.5	85.5	79.8	74	88.6	99.5

Table SI 13: Table of task 3 DiffSBDD full outputs for each protein, with reasons given for failures and percentage of passes in each category

prot	comments	passed	molgen	MOSES	bond__length	bond__angle	int__clash	int__energy	dist__to__prot	dist__to__wat	vol__prot
**Moonshot**		TRUE	97.7	49.1	94	87.4	89	84.9	74.5	100	98.2
**CSAR**		TRUE	96.7	55.7	92.8	86.5	87	86.4	82.3	100	94.5

MolSnapper outputs

Table SI 14: Table of task 1 MolSnapper full outputs for each protein, with reasons given for failures and percentage of passes in each category

**prot**	**comments**	**passed**	**molgen**	**MOSES**	**bond__length**	**bond__angle**	**int__clash**	**int__energy**	**dist__to__prot**	**dist__to__wat**	**vol__prot**
**1**	Step1/3: Did not pass WT distance t-test;	FALSE	96.30	58.50	86.10	81.60	92.50	93.80	28.00	100.00	96.10
**2**	Generation and/or analysis not run;										
**3**	Step3/4: Did not pass hydrophobic re-creation check;	FALSE	100.00	25.27	61.22	74.41	96.23	88.07	99.84	100.00	100.00
**4**	Step2/4: Did not pass t-test with Fe and without Fe;	FALSE	74.40	56.00	91.80	91.60	98.00	85.00	70.20	100.00	99.20
**5**	Generation and/or analysis not run;										
**6**	Step1/3: Did not have sufficient data points;	FALSE	100.00	35.17	83.79	70.34	90.83	97.86	69.11	100.00	100.00
**7**		TRUE	76.50	64.90	85.90	73.80	98.40	87.60	29.00	100.00	95.30
**8**	Step3/3: Did not pass hydrophobic re-creation check;	FALSE	96.40	67.40	98.60	96.60	96.00	92.40	99.00	100.00	99.80

**9**	Step2/4: Did not pass 1pl6 H2O and no H2O hbond and hydrophobic interactions check;Did not contain sufficient 1pl7 data;	FALSE	63.60	57.10	87.30	84.70	98.80	70.40	18.90	96.20	99.80
**10**	Step2/4: Did not pass hbond re-creation check;	FALSE	90.60	62.00	71.20	74.80	95.20	92.80	86.60	100.00	99.80
**11**	Step4/5: Did not pass apo hydrophobic, hbond, saltbridge re-creation checks;	FALSE	71.30	75.10	95.70	91.00	94.60	93.30	61.30	100.00	92.00
**12**	Step2/4: Did not pass hydrohobic, hbond, saltbridge re-creations with Zn check;	FALSE	95.80	21.73	93.33	82.22	97.28	92.10	48.64	100.00	99.51
**13**	Analysis not run or less than 100 compounds generated;										
**14**		TRUE	97.50	59.70	94.00	87.20	96.90	97.50	15.90	100.00	18.20

**15**	Analysis not run or less than 100 compounds generated;										
**16**	Step3/4: Did not pass hbond and hydrophobic re-creations check;	FALSE	93.13	67.33	89.80	89.47	95.67	94.27	36.87	59.53	74.27
**17**	Analysis not run or less than 100 compounds generated;										
**18**	Step2/3: Did not pass hydrophobic re-creation check;	FALSE	94.73	58.93	98.2	95.93	96.47	98.33	6.00	57.13	27.67
**19**		TRUE	54.13	58.33	86.07	87.80	96.67	57.27	43.4	95.33	77.73
**20**	Step2/3: Did not pass apo hydrophobic and hbond re-creation check;	FALSE	58.30	64.00	90.80	86.90	98.00	70.30	32.80	94.10	58.00

Table SI 15: Table of task 2 MolSnapper full outputs for each protein, with reasons given for failures and percentage of passes in each category

prot	comments	passed	molgen	MOSES	bond__length	bond__angle	int__clash	int__energy	dist__to__prot	dist__to__wat	vol__prot
**ITK**	Analysis not run or less than 100 compounds generated;										
**JAK**		TRUE	88.60	42.83	76.12	81.67	94.20	0	61.72	100.00	96.75
**BET**	Analysis not run or less than 100 compounds generated;										
**shik**	Step2/3: Did not pass Asp32 hbond re-creation check;	FALSE	31.80	15.20	63.80	89.80	80.20	51.30	80.30	98.70	100.00

Table SI 16: Table of task 3 MolSnapper full outputs for each protein, with reasons given for failures and percentage of passes in each category

**prot**	**comments**	**passed**	**molgen**	**MOSES**	**bond__length**	**bond__angle**	**int__clash**	**int__energy**	**dist__to__prot**	**dist__to__wat**	**vol__prot**
**Moonshot**		TRUE	66.30	52.90	77.50	85.80	90.90	91.00	91.60	100.00	100.00
**CSAR**		TRUE	88.00	42.50	86.10	90.60	95.00	84.00	28.50	100.00	73.90

AutoGrow4 outputs

Table SI 17: Table of task 1 AutoGrow4 full outputs for each protein, with reasons given for failures and percentage of passes in each category

prot	comments	passed	molgen	MOSES	bond__length	bond__angle	int__clash	int__energy	dist__to__prot	dist__to__wat	vol__prot
**1**		TRUE	100.00	27.00	100.00	100.00	100.00	99.0	100.00	100.00	100.00
**2**	Analysis not run or less than 100 compounds generated;										
**3**	Analysis not run or less than 100 compounds generated;										
**4**	Analysis not run or less than 100 compounds generated;										
**5**	Analysis not run or less than 100 compounds generated;										
**6**		TRUE	100.00	50.47	100.00	100.00	100.00	99.07	100.00	100.00	100.00
**7**	Analysis not run or less than 100 compounds generated;										
**8**	Analysis not run or less than 100 compounds generated;										

**9**	Step2/4: Did not pass 1pl6 H2O and no H2O hbond and hydrophobic interactions check;Did not contain sufficient 1pl7 data;	FALSE	100.00	36.40	100.00	100.00	100.00	92.51	100.00	100.00	100.00
**10**	Generation and/or analysis not run;										
**11**	Generation and/or analysis not run;										
**12**	Analysis not run or less than 100 compounds generated;										
**13**	Step1/6: Did not contain sufficient data points;	FALSE	100.00	31.22	100.00	100.00	100.00	95.48	100.00	100.00	100.00
**14**		TRUE	100.00	46.32	100.00	100.00	100.00	94.74	100.00	100.00	100.00
**15**	Analysis not run or less than 100 compounds generated;										

**16**	Step3/4: Did not pass hbond and hydrophobic re-creations check;	FALSE	100.00	31.61	100.00	100.00	100.00	91.61	100.00	100.00	100.00
**17**	Step5/7: Did not contain sufficient data points;	FALSE	100.00	47.59	100.00	100.00	100.00	91.03	100.00	100.00	100.00
**18**	Step2/3: Did not pass hydrophobic re-creation check;	FALSE	100.00	52.78	100.00	100.00	100.00	97.92	100.00	100.00	100.00
**19**	Step2/2: Did not pass having more acidic groups with Ca;	FALSE	100.00	63.96	100.00	100.00	100.00	91.88	100.00	100.00	100.00
**20**	Analysis not run or less than 100 compounds generated;										

Table SI 18: Table of task 2 AutoGrow4 full outputs for each protein, with reasons given for failures and percentage of passes in each category

**prot**	**comments**	**passed**	**molgen**	**MOSES**	**bond__length**	**bond__angle**	**int__clash**	**int__energy**	**dist__to__prot**	**dist__to__wat**	**vol__prot**
**ITK**	Could not run task test, division by zero;										
**JAK**	Step1/2: Did not contain sufficient data points;	FALSE	100.00	43.35	100.00	100.00	100.00	94.22	100.00	100.00	100.00
**BET**	Step1/4: Did not contain sufficient data points;	FALSE	100.00	37.76	100.00	100.00	100.00	91.84	100.00	100.00	100.00
**shik**	Analysis not run or less than 100 compounds generated;										

Table SI 19: Table of task 3 AutoGrow4 full outputs for each protein, with reasons given for failures and percentage of passes in each category

prot	comments	passed	molgen	MOSES	bond__length	bond__angle	int__clash	int__energy	dist__to__prot	dist__to__wat	vol__prot
**Moonshot**	Analysis not run or less than 100 compounds generated;										
**CSAR**		TRUE	99.90	38.50	100.00	100.00	100.00	93.70	100.00	100.00	100.00

LigBuilderV3 outputs

Table SI 20: Table of task 1 LigBuilderV3 full outputs for each protein, with reasons given for failures and percentage of passes in each category

**prot**	**comments**	**passed**	**molgen**	**MOSES**	**bond__length**	**bond__angle**	**int__clash**	**int__energy**	**dist__to__prot**	**dist__to__wat**	**vol__prot**
**1**	Generation and/or analysis not run;										
**2**	Generation and/or analysis not run;										
**3**	Step1/4: Did not have sufficient data points;	FALSE	99.9	41.86	99.9	99.9	95.8	99.8	83.42	99.9	98.8
**4**	Generation and/or analysis not run;										
**5**	Generation and/or analysis not run;										
**6**	Could not run task test, '6ryp__gen__mol__min__dist';										
**7**	Generation and/or analysis not run;										
**8**	Step2/3: Did not pass hbond re-creation check;	FALSE	100	60.6	100	100	99.2	100	98.3	100	100

**9**	Step1/4: Did not contain sufficient 1pl6 data;Did not contain sufficient 1pl7 data;	FALSE	99.21	67.86	99.21	99.21	97.62	99.11	95.93	99.21	99.11
**10**	Generation and/or analysis not run;										
**11**	Generation and/or analysis not run;										
**12**	Generation and/or analysis not run;										
**13**	Generation and/or analysis not run;										
**14**	Generation and/or analysis not run;										
**15**	Generation and/or analysis not run;										

**16**	Could not run task test, '5yjw _allosteric _gen _mol _com _dist';										
**17**	Could not run task test, 'NoneType' object is not iterable;										
**18**	Generation and/or analysis not run;										
**19**	Generation and/or analysis not run;										
**20**	Step1/3: Did not contain sufficient data points; Step2/3: Did not pass apo hydrophobic and hbond re-creation check;	FALSE	99.81	61.12	99.81	99.81	99.44	99.81	36.07	37.38	37.38

Table SI 21: Table of task 2 LigBuilderV3 full outputs for each protein, with reasons given for failures and percentage of passes in each category

prot	comments	passed	molgen	MOSES	bond__length	bond__angle	int__clash	int__energy	dist__to__prot	dist__to__wat	vol__prot
**ITK**	Could not run task test, division by zero;										
**JAK**	Analysis not run or less than 100 compounds generated;										
**BET**	Could not run task test, division by zero;										
**shik**	Step2/3: Did not pass Asp32 hbond re-creation check;	FALSE	100	47.5	100	100	99.6	100	71.7	72.9	73.3

Table SI 22: Table of task 3 LigBuilderV3 full outputs for each protein, with reasons given for failures and percentage of passes in each category

**prot**	**comments**	**passed**	**molgen**	**MOSES**	**bond__length**	**bond__angle**	**int__clash**	**int__energy**	**dist__to__prot**	**dist__to__wat**	**vol__prot**
**Moonshot**	Step2/3: Did not pass hbond re-creation check;	FALSE	100	51.1	100	100	93.5	99.8	92.8	100	100
**CSAR**	Step2/3: Did not pass hbond re-creation check;	FALSE	100	52.4	100	100	95.5	99.6	93.5	100	99.9

ITK PLIFs

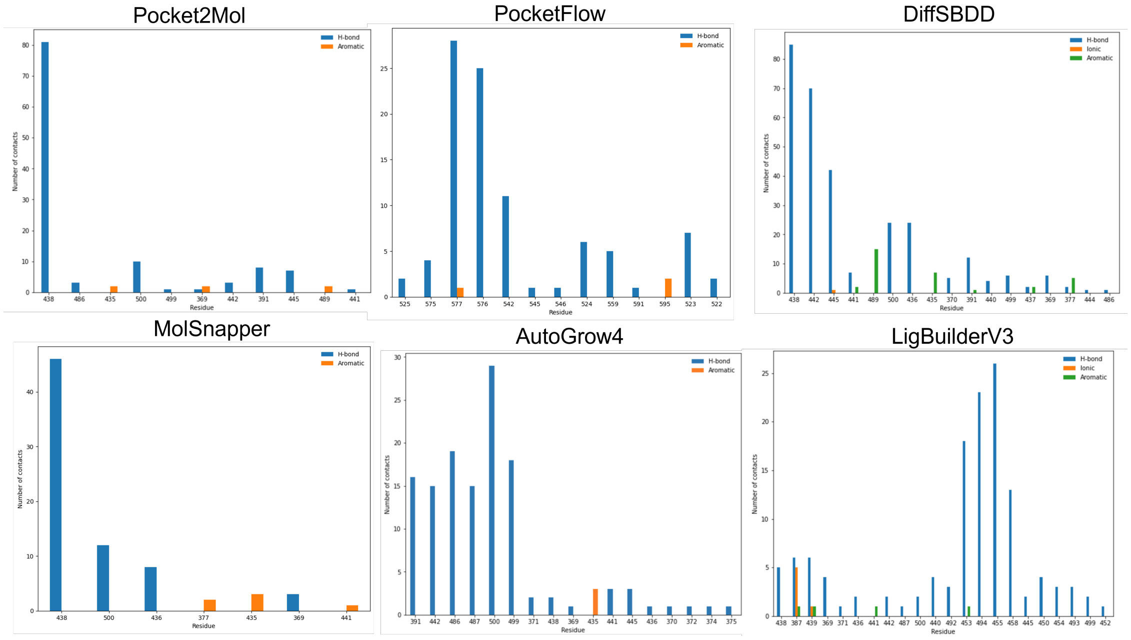

Figure SI 4: Protein-ligand interaction fingerprints (PLIFs) generated in MOE for 100 randomly selected compounds generated for ITK PDB 4l7s, where only one crystal structure hydrogen bond interaction is observed at residue 438

Synthesizability

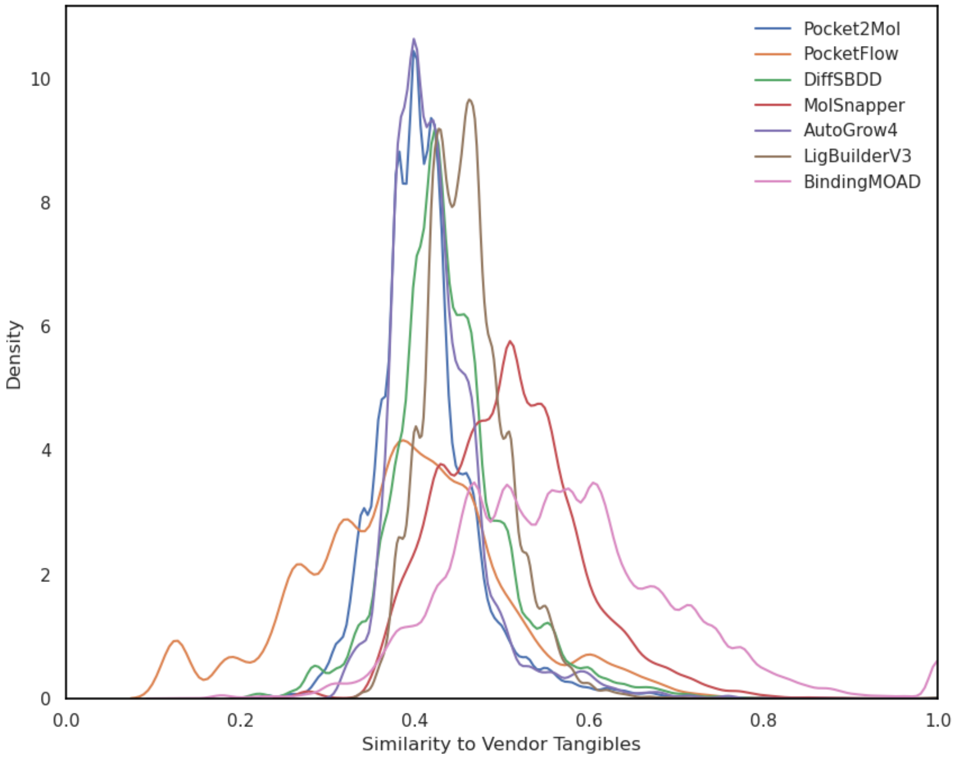

Figure SI 5: The distributions of Tanimoto similarities computed with the RDKit default substructure-based fingerprint with 2048 bits for each generator compared to the GSK Vendor Tangibles dataset

Property Distributions

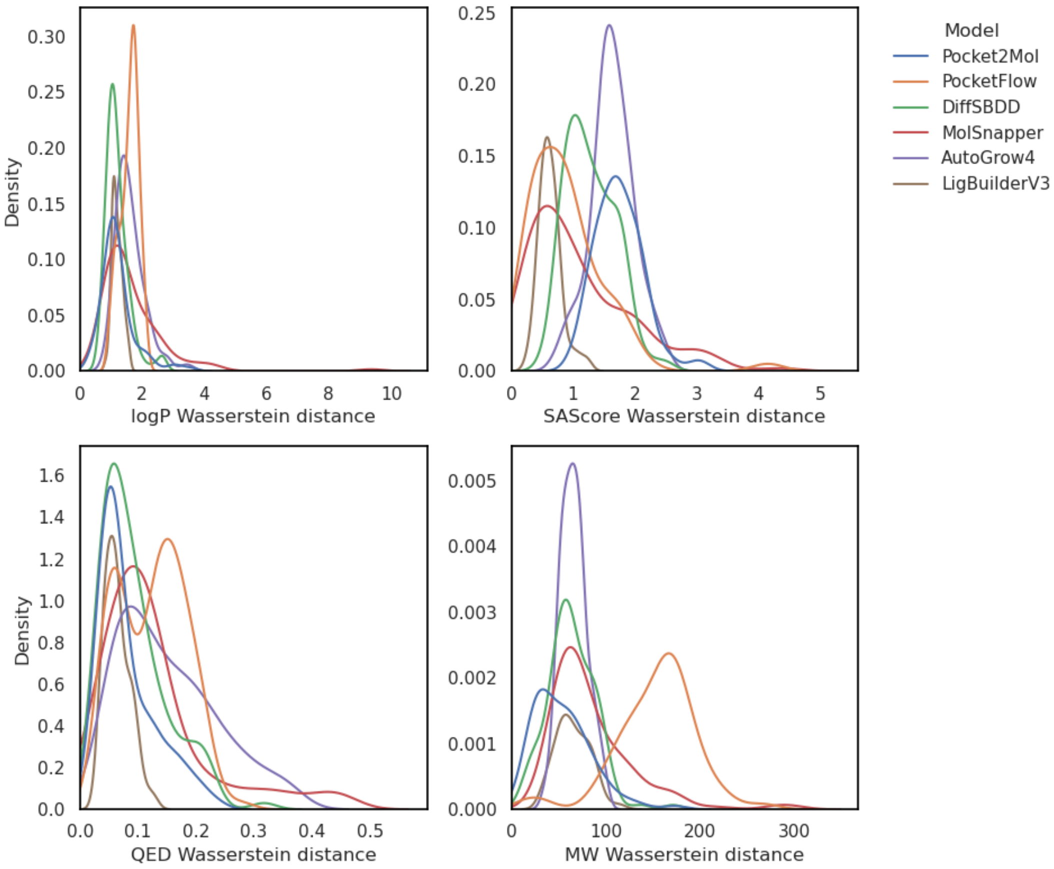

Figure SI 6: Distributions showing the Wasserstein divergence of generated compounds compared to BindingMOAD for logP, SAScore, QED, and molecular weight

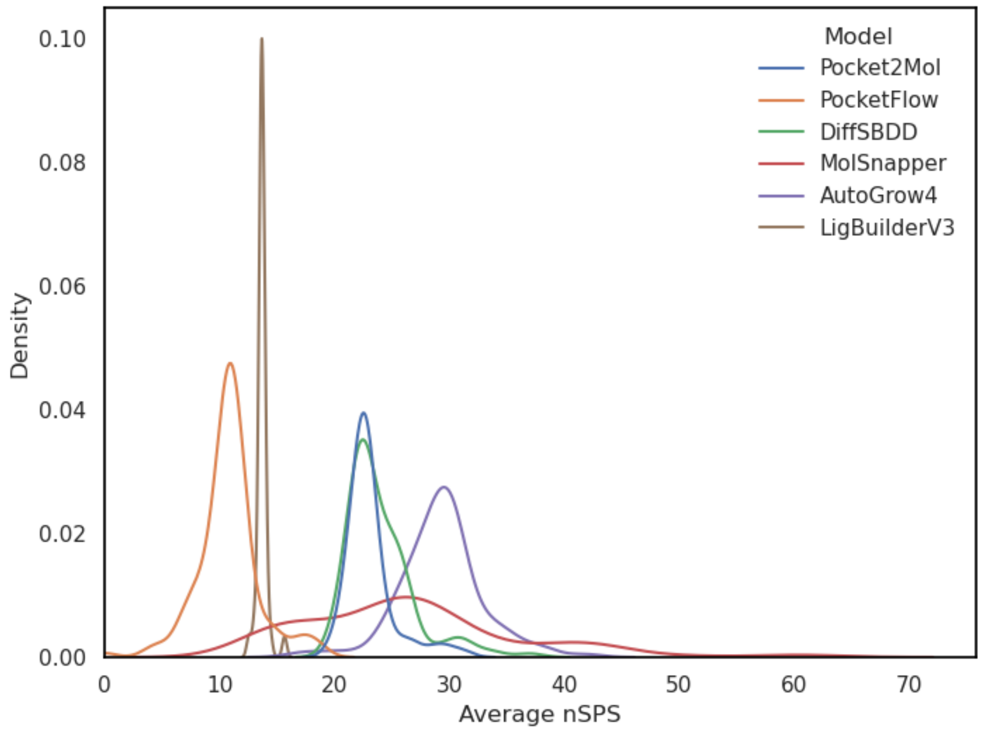

Figure SI 7: Normalized spacial score distributions of generated compounds for each generative model

Pocket2Mol re-training

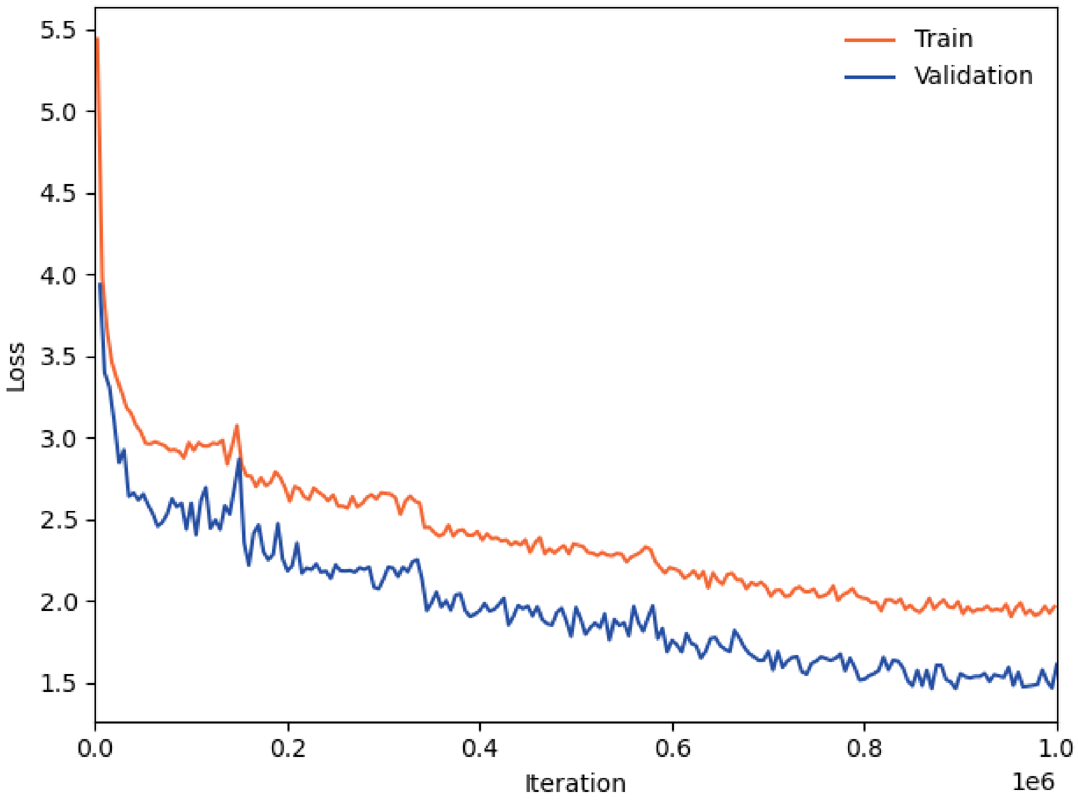

Figure SI 8: Pocket2Mol train and validation loss curves per iteration of sequential molecule generation for the BindingMOAD dataset, ran for a total of 1,000,000 iterations

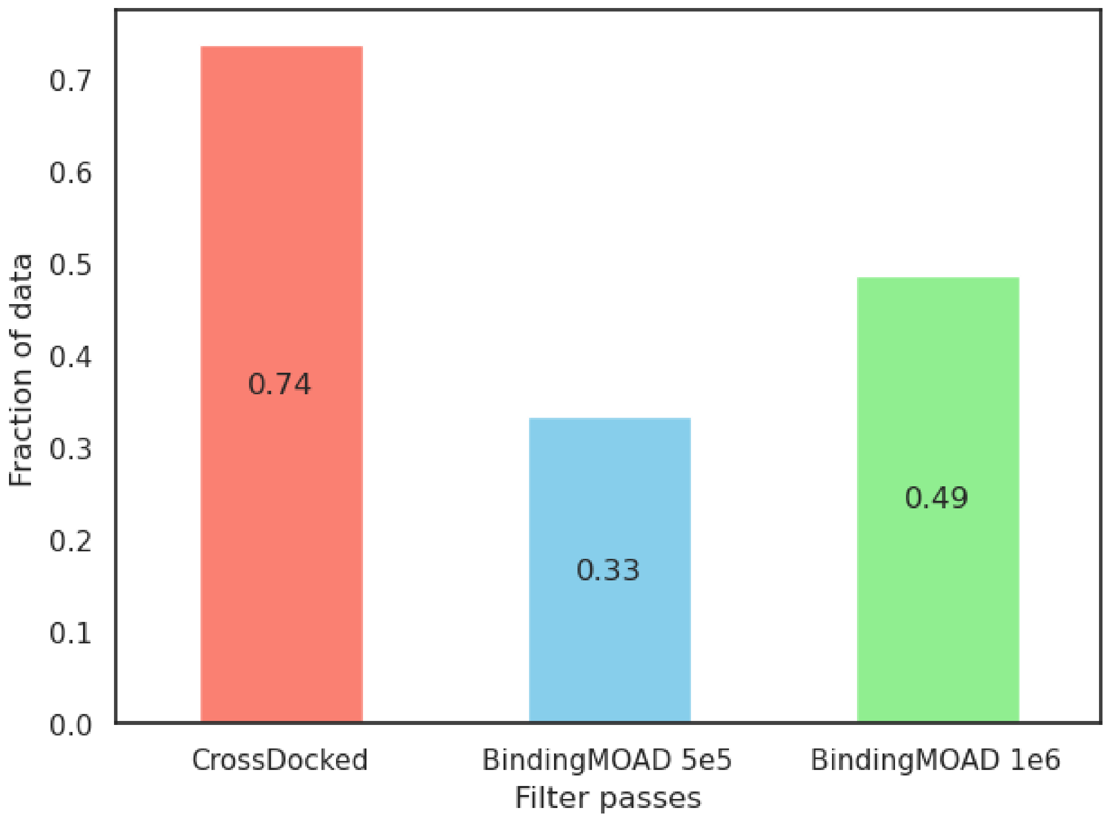

Figure SI 9: A bar chart showing the fraction of Pocket2Mol-generated compounds for Task 2 (ITK, LCK, AurB selectivity) passing allene and fused ring checks when trained on the original CrossDocked2020, re-trained on the BindingMOAD dataset for 500,000 iterations, and 1,000,000 iterations

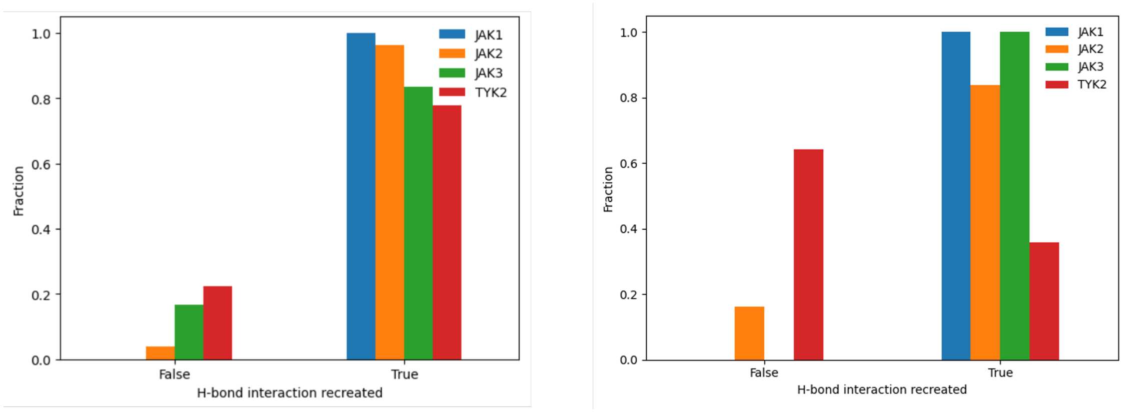

Figure SI 10: Pocket2Mol interaction re-creations for pan-JAK task: (Left) BindingMOAD re-trained for 500,000 iterations. (Right) BindingMOAD re-trained for 1,000,000 iterations

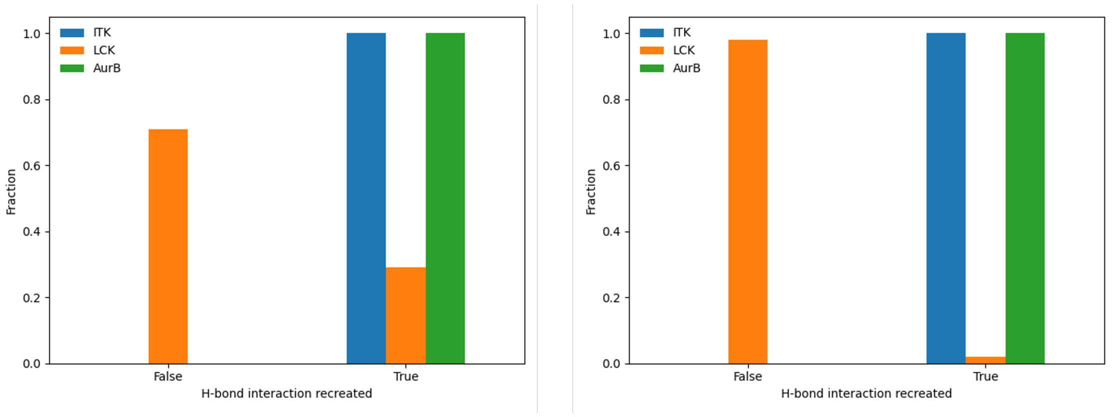

Figure SI 11: Pocket2Mol interaction re-creations for ITK selectivity task: (Left) BindingMOAD re-trained for 500,000 iterations. (Right) BindingMOAD re-trained for 1,000,000 iterations

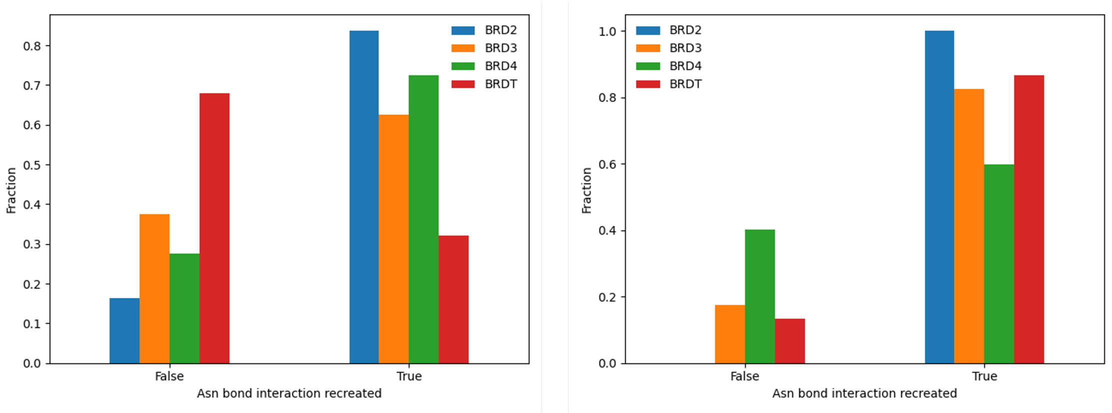

Figure SI 12: Pocket2Mol interaction re-creations for pan-BET task: (Left) BindingMOAD re-trained for 500,000 iterations. (Right) BindingMOAD re-trained for 1,000,000 iterations

Conformation RMSDs

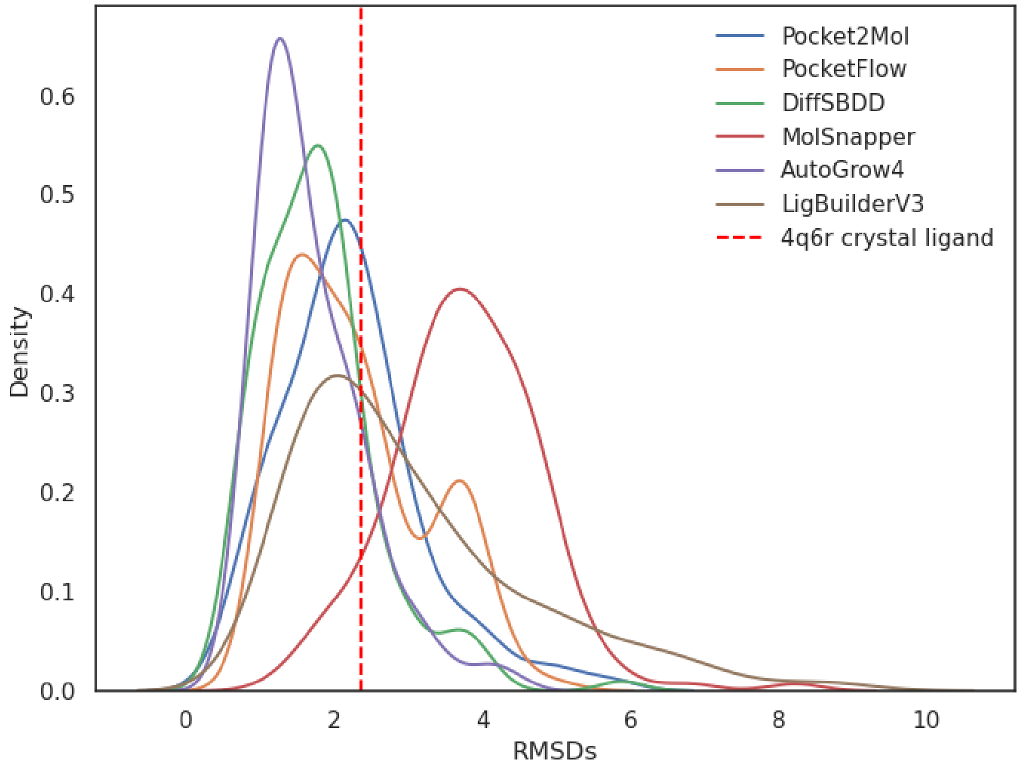

Figure SI 13: The RMSDs, in Angstroms, pre- and post- Embrace minimization for each generative method. The dashed red vertical line indicates the crystal ligand RMSD at 2.37 Å.

Mean and standard deviations of re-created interactions

Table SI 23: The mean fraction of re-created interactions per task for each evaluated method after compound filtering through MOSES. The standard deviation across proteins in each task are shown in brackets.

	**Task 1**	**Task 2**	**Task 3**
**Pocket2Mol**	0.249 (0.163)	0.714 (0.237)	0.242 (0.0641)
**PocketFlow**	0.0848 (0.141)	0.363 (0.415)	0.00 (0.00)
**DiffSBDD**	0.472 (0.203)	0.810 (0.161)	0.584 (0.0118)
**MolSnapper**	0.474 (0.319)	0.678 (0.295)	0.695 (0.147)
**AutoGrow4**	0.234 (0.174)	0.400 (0.195)	0.185 (0.102)
**LigBuilderV3**	0.0529 (0.0818)	0.225 (0.294)	0.0252 (0.0252)

References

(1) Peng, X.; Luo, S.; Guan, J.; Xie, Q.; Peng, J.; Ma, J. Pocket2Mol: Efficient Molecular Sampling Based on 3D Protein Pockets. Preprint available at https://arxiv.org/abs/2205.07249 (2022).

(2) Jiang, Y.; Zhang, G.; You, J.; Zhang, H.; Yao, R.; Xie, H.; Zhang, L.; Xia, Z.; Dai, M.; Wu, Y.; Li, L.; Yang, S. PocketFlow is a data-and-knowledge-driven structure-based molecular generative model. *Nature Machine Intelligence* **2024**, *6*, 326–337.

(3) Schneuing, A.; Harris, C.; Du, Y.; Didi, K.; Jamasb, A.; Igashov, I.; Du, W.; Gomes, C.; Blundell, T. L.; Lio, P.; Welling, M.; Bronstein, M.; Correia, B. Structure-based drug design with equivariant diffusion models. *Nature Computational Science* **2024**, *4*, 899–909.

(4) O'Boyle, N. M.; Banck, M.; James, C. A.; Morley, C.; Vandermeersch, T.; Hutchison, G. R. Open Babel: An open chemical toolbox. *Journal of Cheminformatics* **2011**, *3*, 33.

(5) Ziv, Y.; Imrie, F.; Marsden, B.; Deane, C. M. MolSnapper: Conditioning Diffusion for Structure-Based Drug Design. *Journal of Chemical Information and Modeling* **2025**, *65*, 4263–4273.

(6) Peng, X.; Guan, J.; Liu, Q.; Ma, J. MolDiff: Addressing the Atom-Bond Inconsistency Problem in 3D Molecule Diffusion Generation. Preprint available at https://arxiv.org/abs/2305.07508 (2023).

(7) Zou, X.; Zhao, X.; Liò, P.; Zhao, Y. Will More Expressive Graph Neural Networks do Better on Generative Tasks? Preprint available at https://arxiv.org/abs/2308.11978 (2023).

(8) Spiegel, J. O.; Durrant, J. D. AutoGrow4: an open-source genetic algorithm for de novo drug design and lead optimization. *Journal of Cheminformatics* **2020**, *12*, 25.

(9) Chakravorty, S. J.; Chan, J.; Greenwood, M. N.; Popa-Burke, I.; Remlinger, K. S.; Pickett, S. D.; Green, D. V.; Fillmore, M. C.; Dean, T. W.; Luengo, J. I.; Macarrón, R. Nuisance Compounds, PAINS Filters, and Dark Chemical Matter in the GSK HTS Collection. *SLAS Discovery* **2018**, *23*, 532–544.

(10) Ropp, P. J.; Spiegel, J. O.; Walker, J. L.; Green, H.; Morales, G. A.; Milliken, K. A.; Ringe, J. J.; Durrant, J. D. Gypsum-DL: an open-source program for preparing small-molecule libraries for structure-based virtual screening. *Journal of Cheminformatics* **2019**, *11*, 34.

(11) Trott, O.; Olson, A. J. AutoDock Vina: Improving the speed and accuracy of docking with a new scoring function, efficient optimization, and multithreading. *Journal of Computational Chemistry* **2009**, *31*, 455–461.

(12) Yuan, Y.; Pei, J.; Lai, L. LigBuilder V3: A Multi-Target de novo Drug Design Approach. *Frontiers in Chemistry* **2020**, *8*, 142.

(13) Yuan, Y.; Pei, J.; Lai, L. LigBuilder 2: A Practical de Novo Drug Design Approach. *Journal of Chemical Information and Modeling* **2011**, *51*, 1083–1091.

(14) Wang, R.; Gao, Y.; Lai, L. LigBuilder: A Multi-Purpose Program for Structure-Based Drug Design. *Journal of Molecular Modeling* **2000**, *6*, 498–516.

(15) Ballinger, E. et al. Opposing reactions in coenzyme A metabolism sensitize Mycobacterium tuberculosis to enzyme inhibition. *Science* **2019**, *363*, eaau8959.

(16) Thoma, R.; Schulz-Gasch, T.; D'Arcy, B.; Benz, J.; Aebi, J.; Dehmlow, H.; Hennig, M.; Stihle, M.; Ruf, A. Insight into steroid scaffold formation from the structure of human oxidosqualene cyclase. *Nature* **2004**, *432*, 118–122.

(17) Shi, K.; Carpenter, M. A.; Kurahashi, K.; Harris, R. S.; Aihara, H. Crystal Structure of the DNA Deaminase APOBEC3B Catalytic Domain. *Journal of Biological Chemistry* **2015**, *290*, 28120–28130.

(18) Xu, S.; Mueser, T. C.; Marnett, L. J.; Funk, M. O. Crystal Structure of 12-Lipoxygenase Catalytic-Domain-Inhibitor Complex Identifies a Substrate-Binding Channel for Catalysis. *Structure* **2012**, *20*, 1490–1497.

(19) McGrath, A. P.; Hilmer, K. M.; Collyer, C. A.; Shepard, E. M.; Elmore, B. O.; Brown, D. E.; Dooley, D. M.; Guss, J. M. Structure and Inhibition of Human Diamine Oxidase. *Biochemistry* **2009**, *48*, 9810–9822.

(20) Olatunji, S.; Yu, X.; Bailey, J.; Huang, C.-Y.; Zapotoczna, M.; Bowen, K.; Remškar, M.; Müller, R.; Scanlan, E. M.; Geoghegan, J. A.; Olieric, V.; Caffrey, M. Structures of lipoprotein signal peptidase II from Staphylococcus aureus complexed with antibiotics globomycin and myxovirescin. *Nature Communications* **2020**, *11*, 140.

(21) Homan, K. T.; Wu, E.; Wilson, M. W.; Singh, P.; Larsen, S. D.; Tesmer, J. J. G. Structural and Functional Analysis of G Protein-Coupled Receptor Kinase Inhibition by Paroxetine and a Rationally Designed Analog. *Molecular Pharmacology* **2013**, *85*, 237–248.

(22) Weiler, S.; Braendlin, N.; Beerli, C.; Bergsdorf, C.; Schubart, A.; Srinivas, H.; Oberhauser, B.; Billich, A. Orally Active 7-Substituted (4-Benzylphthalazin-1-yl)-2-methylpiperazin-1-yl]nicotinonitriles as Active-Site Inhibitors of Sphingosine 1-Phosphate Lyase for the Treatment of Multiple Sclerosis. *Journal of Medicinal Chemistry* **2014**, *57*, 5074–5084.

(23) Pauly, T. A. et al. X-Ray Crystallographic and Kinetic Studies of Human Sorbitol Dehydrogenase. *Structure* **2003**, *11*, 1071–1085.

(24) Nitanai, Y.; Satow, Y.; Adachi, H.; Tsujimoto, M. Crystal Structure of Human Renal Dipeptidase Involved in β-Lactam Hydrolysis. *Journal of Molecular Biology* **2002**, *321*, 177–184.

(25) Pallarès, I.; Fernández, D.; Comellas-Bigler, M.; Fernández-Recio, J.; Ventura, S.; Avilés, F. X.; Bode, W.; Vendrell, J. Direct interaction between a human digestive protease and the mucoadhesive poly(acrylic acid). *Acta Crystallographica Section A* **2008**, *64*, 784–791.

(26) Yang, Y.; Yu, Y.; Li, X.; Li, J.; Wu, Y.; Yu, J.; Ge, J.; Huang, Z.; Jiang, L.; Rao, Y.; Yang, M. Target Elucidation by Cocrystal Structures of NADH-Ubiquinone Oxidoreductase of Plasmodium falciparum (PfNDH2) with Small Molecule To Eliminate Drug-Resistant Malaria. *Journal of Medicinal Chemistry* **2017**, *60*, 1994–2005.

(27) Ebenhoch, R.; Prinz, S.; Kaltwasser, S.; Mills, D. J.; Meinecke, R.; Rübbelke, M.; Reinert, D.; Bauer, M.; Weixler, L.; Zeeb, M.; Vonck, J.; Nar, H. A hybrid approach reveals the allosteric regulation of GTP cyclohydrolase I. *Proceedings of the National Academy of Sciences of the United States of America* **2020**, *117*, 31838–31849.

(28) Yamashita, T.; Inaoka, D. K.; Shiba, T.; Oohashi, T.; Iwata, S.; Yagi, T.; Kosaka, H.; Miyoshi, H.; Harada, S.; Kita, K.; Hirano, K. Ubiquinone binding site of yeast NADH

dehydrogenase revealed by structures binding novel competitive- and mixed-type inhibitors. *Scientific Reports* **2018**, *8*, 2427.

(29) Carl, A. G.; Harris, L. D.; Feng, M.; Nordstrøm, L. U.; Gerfen, G. J.; Evans, G. B.; Silakov, A.; Almo, S. C.; Grove, T. L. Narrow-Spectrum Antibiotic Targeting of the Radical SAM Enzyme MqnE in Menaquinone Biosynthesis. *Biochemistry* **2020**, *59*, 2562–2575.

(30) Haynes, C. A.; Koder, R. L.; Miller, A.-F.; Rodgers, D. W. Structures of Nitroreductase in Three States. *Journal of Biological Chemistry* **2002**, *277*, 11513–11520.

(31) Janocha, S.; Carius, Y.; Hutter, M.; Lancaster, C. R. D.; Bernhardt, R. Crystal Structure of CYP106A2 in Substrate-Free and Substrate-Bound Form. *ChemBioChem* **2016**, *17*, 852–860.
